# Micro-architecture design exploration template for AutoML case study on SqueezeSEMAuto

**DOI:** 10.1038/s41598-023-37682-0

**Published:** 2023-06-30

**Authors:** Chantana Chantrapornchai, Supasit Kajkamhaeng, Phattharaphon Romphet

**Affiliations:** Department of Computer Engineering, Faculty of Engineering, Kasetsart University, Bangkok, Thailand

**Keywords:** Software, Scientific data, Computational science, Information technology

## Abstract

Convolutional Neural Network (CNN) models have been commonly used primarily in image recognition tasks in the deep learning area. Finding the right architecture needs a lot of hand-tune experiments which are time-consuming. In this paper, we exploit an AutoML framework that adds to the exploration of the micro-architecture block and the multi-input option. The proposed adaption has been applied to SqueezeNet with SE blocks combined with the residual block combinations. The experiments assume three search strategies: Random, Hyperband, and Bayesian algorithms. Such combinations can lead to solutions with superior accuracy while the model size can be monitored. We demonstrate the application of the approach against benchmarks: CIFAR-10 and Tsinghua Facial Expression datasets. The searches allow the designer to find the architectures with better accuracy than the traditional architectures without hand-tune efforts. For example, CIFAR-10, leads to the SqueezeNet architecture using only 4 fire modules with 59% accuracy. When exploring SE block insertion, the model with good insertion points can lead to an accuracy of 78% while the traditional SqueezeNet can achieve an accuracy of around 50%. For other tasks, such as facial expression recognition, the proposed approach can lead up to an accuracy of 71% with the proper insertion of SE blocks, the appropriate number of fire modules, and adequate input merging, while the traditional model can achieve the accuracy under 20%.

## Introduction

Designing convolutional neural network (CNN) models require a lot of extensive experiments. Besides exploring the machine learning parameters such as learning rates, and momentum, also, for each layer, different hyperparameters should be considered. For example, for the convolution layer, one needs to explore the convolution filter sizes, the number of strides, an initialization approach, etc., for the dropout layers, a dropout rate can be varied, and for the pooling layers, the pooling sizes and pooling operations, e.g., max, average, global average are considered. The explorations of these parameters are time consuming and need to be done systematically to alleviate the model construction process.

Beside the exploration in each layer design, one needs to find the right structure for the specific recognition task. Searching for all possible structures is very computational-intensive and consumes resources. If the specific forms of the architectures are allowed, searching for the right connections may be possible within acceptable accuracy and time.

In this research, we study the methodology for automatic exploration of the micro-architecture types. If we are given the baseline architecture, our approach enables the search for model modification automatically to obtain good accuracy. We demonstrate the approach using the vanilla SqueezeNet^1^ since it is a small architecture and it can still be expanded. The proposed approach will enable the neural network designer to explore models to answer typical design questions such as:

- What is the number of convolutions blocks that are needed?
- The number of features that should be used and how they are merged?
- Should the skip connection be applied and where to apply?
- What if some optimization block is inserted and where to inserted properly?

The flexible design of SqueezeNet as a prototype, called *SqueezeNetSEMAuto* is presented. It is the traditional SqueezeNet with a flexible length, the selected SE block insertion and skip connection, and the selected merging operation for more than one input. It also can be integrated with the hyperparameter exploration for each layer. The source of the prototype models is available at github https://github.com/cchanra/SqueezeNetSEMAuto.

The structure of the paper is as follows. Next, we introduce the background and literature review. In Section “Design methodology”, the methodology of flexible design is described. Section “Experiments” presents the experimental methods and results. Finally, Section “Other applications” concludes the work and explains future work.

## Background

In this section, we introduce the convolution neural network and some previous models. Next, the complexity in various levels of model exploration is explained. At last, we present the literature review of model exploration.

### Convolutional neural networks

Convolutional Neural Networks (CNN)^2^ are designed based on convolution operations. It has been applied to many high-impact image processing tasks such as medical imaging^3,4^, self-driving car^5,6^, etc. They have convolutional layers connected in some manners. The layer utilizes the convolution operation with using the fixed-size filter to perform such operation with the matrices or tensors. The operations involved are multiplications and additions.

The typical CNN model consists of the following layers: convolution, pooling, dropout, activation, fully-connected, etc. These layers are connected in certain manners. Convolution layers have important parameters such as the filter, stride, padding sizes. The filter and stride sizes imply the output feature map sizes and the number of consecutive convolution layers implies the size of receptive fields.

Pooling layers are used to reduce the feature size. They also can help select outstanding features. They have the typical parameters which are such as the pooling and stride sizes. Pooling operations are e.g. max, average, and global average pooling. The various combinations of the convolutions and pooling cause the different feature map output values and sizes.

The dropout layer is used for the purpose of overfitting elimination. Its typical parameter is the dropout rate ranging between 0 and 1. The dropout rate needs to be examined as well.

The activation function is applied to change the output values. Activation functions are selected depending on the tasks, e.g., ReLU, LeakyReLu, Sigmoid, Tanh, and many others. The fully connected layers (FC) are usually attached at last for classification outputs. The number of fully connected layers can be more than one depending on the design and the number of classification outputs.

Besides the above-mentioned aspects which are called *hyperparameters* of CNN, the learning rate, and optimization function can also affect the high accuracy. The common learning rate values are 0.1, 0.01, 0.001, or 0.0001. The very small learning rate leads to a slow convergence and may get stuck in local minima while using a large learning rate can skip the global optimal values. Sometimes, the learning rate is also described as a function. The optimization functions can be Adam, RMSProp, Stochastic Gradient Descent (SGD), Adagrad, etc. Different optimization yields different gradients and directions leading to the optimal weights.

### State-of-the-arts architectures

Various designed modules for CNN are proposed to lengthen the network to increase accuracy. Some modules can reduce the computation and the model size while maintaining accuracy.

One of the popular pre-trained models such as AlexNet^7^ was developed by Krizhevsky et.al. It was trained by ImageNet dataset in ILSVRC-2010 and ILSVRC-2012, with 1.2 million images in 1000 categories. The architecture contains eight learnable layers five convolutional and three fully connected (called *fc6, fc7, fc8*). The first five layers are convolutional and the remaining three are fully connected.

The reference CaffeNet^8^ is a similar version of AlexNet, except that the max pooling layer precedes the local response normalization (LRN) to reduce memory usage. GoogLeNet^9^ is a deep convolutional neural network structure. It was used as a classification and detection tool in ILSVRC14 with the goal to work with small data sets and use small computing power and memory. It employs an inception module that simultaneously computes 1×1, 3×3, and 5×5 convolutions, enabling the selection of proper filter sizes automatically. It was trained in ILSVRC 2014 challenge to classify the image into one of 1000 leaf-node categories. ImageNet dataset consists of over 1.2 million training images, 50,000 validation and 100,000 testing images.

SqueezeNet^1^ aims to improve AlexNet efficiency while holding the same level of accuracy. The minimized CNN has advantages: saving communication time between the server and the clients for over-the-air updates, and feasibility for embedded-device deployment. SqueezeNet utilizes the methods such as reducing filter sizes, reducing the number of input channels, and delaying downsampling. SqueezeNet was trained on ILSVRC2012 ImageNet. The design focuses on achieving a smaller model size while keeping the same accuracy as AlexNet.

VGG net improved the accuracy by adding more convolutional layers and removing LRN layers. It was trained on ImageNet ILSVRC-2014^10^. The model has various numbers of layers: 11, 13, 16, and 19 layers, making the model parameters vary between 133 and 144 million. It was trained on ILSVRC2012 ImageNet (1.3 million images, with 50K validation images, and 100K testing images).

ResNet was one of the very first models that contain many layers. Particularly, it consists of many convolutional blocks consecutively. The convolutional block forms a residual block designed to solve the gradient vanishing or exploding problem. The network won the ILSVRC competition in 2015. It has variations such as ResNet50, ResNet101, ResNet152. It may be combined with modules in GoogLeNet, known as GoogleResNet etc.

SENet was proposed^11^, based on two subsequence modules, *Squeeze* and *Excitation*. It has the squeeze and excite operations. The squeeze operation performs the combining of feature maps across the dimension $H\times W$ to obtain a channel descriptor. The excitation operation captures the channel dependency and learns the relationships between channels. It performs the activation of the excitation on each channel.

To utilize these above-pre-trained models, transfer learning is a common approach that transfers the knowledge from the source model to the target model^12^. For image applications, image features such as edges, and shapes are learned in the early layers. They are used in the later fully connected layers which are supposed to be fine-tuned for specific tasks. It is useful when the target data set size is smaller than the source data set and when the nature of target images is similar to the source images. The closer they are, the fewer tune layers there should be. With pre-trained models, a small learning rate should be used for pre-trained models so as not to skip unlearned features.

### CNN architecture design

The above typical CNN architectures need to be adjusted when they are applied to a new dataset. At a small scale, one needs to fine-tune the hyperparameters of each layer. At the medium scale, the architecture needs to be adjusted. For example, adding the connection to merge the features from different scales. Some convolutional blocks may be added to reduce the feature map size. At a large scale, the whole architecture can be changed, for example, by changing to use the transformer or sequence-to-sequence.

In this paper, the change in the small scale is called *hyperparameter tuning*. In the medium scale, the connection structures are called *micro-architecture*. From the previous work^13^, using transfer learning to fine-tune to the new task while adopting conventional different architecture does not lead to a significant change in model accuracy. Thus, we are interested in the changes in the micro-architecture level. The adjustment in this level can lead to accuracy improvement and optimal parameters suitable for a specific classification task. The hyperparameter is possible to explore during the micro-architecture search. This sometimes is called, *CNN optimization*.

At the *micro-architecture* level, the choices of using different kinds of layers (e.g. convolutional layers, pooling layers, classification layers) are explored. These layers may be combined into the module for certain purposes. In the module, the convolutional layers certainly play the dominant role in hierarchically extracting meaningful features. As a result, the effective optimization of micro-architecture primarily relates to utilizing the different types of modules to contribute the accuracy while maintaining the network size.

**Figure 1:**
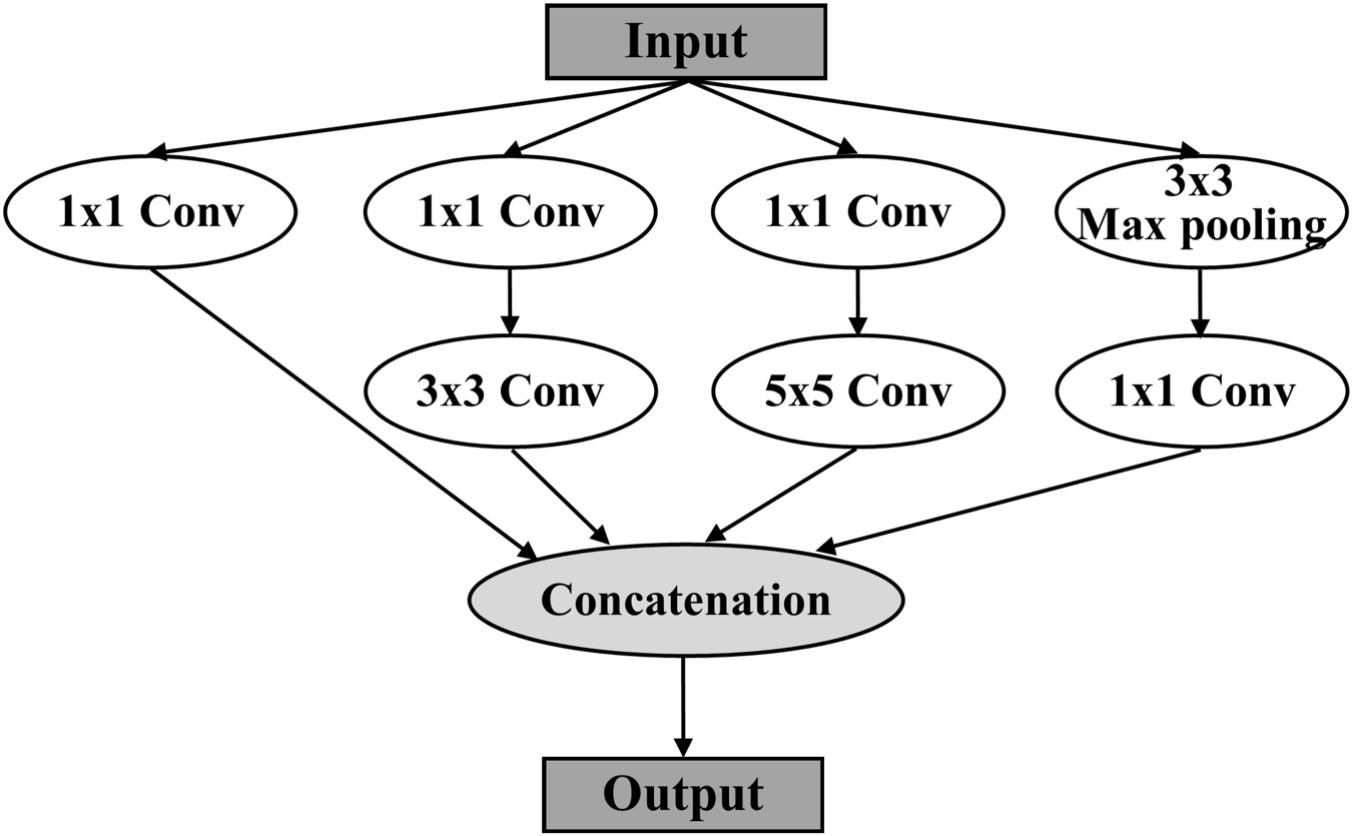
Inception module example^9^.

*Inception module* It is based on GoogLeNet^9^. The purpose of the module is to increase the model depth and make the network wider to allow parallel computation and increase accuracy. The module factorizes the large convolution into smaller ones to reduce the total number of computations and model size. For example, Inception-V1 contains 1x1, 3x3, and 5x5 convolutions, 3x3 max pooling computing simultaneously, and concatenates the results from them together. Figure 1 is an example of the first version of the Inception module. The simultaneous computation of these convolutions can speed up the training time significantly although the network is very deep. The use of various filter sizes enables the selection of proper filter sizes automatically. In Inception-V2, the module is made wider with several small filters, 3x3, 1x3, 3x1, etc. The filter 1x1’s are used to reshape the feature maps and change the dimensionality. The variation of the modules depends on how to factor convolutions to increase the depth and accuracy. GoogLeNet’s training time is faster than other previous networks.

**Figure 2:**
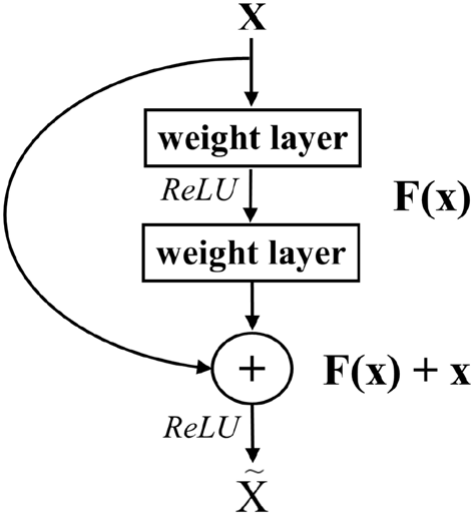
Residual block^14^.

*Residual block* used in ResNet^14^, proposed as shown in Fig. 2 to make the network deeper. In particular, the mapping $F(x)+x$, called *identity mapping* is created and the feature output from previous layers can be transferred. *F*(*x*) is called *residual learning* operation which may be some convolution layers. During the learning, $F(x)+x$ is approximate as well as *F*(*x*). The idea is to solve the problem of accuracy degrading when increasing the depth of the network. This is depicted by the shortcut edge shown in Fig. 2.

Making it deeper this way helps prevent overfitting and exposes more opportunities to improve accuracy by gradually tweaking the model into the underlying function instead of only skipping unneeded layers. The depth of the model is maintained by doing the exact identity mapping for the following layers by adjusting their weights of the residual function to zero. On the other hand, the residual function alleviates a little remaining error of prediction by finding the optimal function which is closer to the identity mapping.

*Skip connection* Highway networks^15^ enables the flow of intermediate data on the previous layers by using the skip connections across the sequence of following layers instead of only the layer-by-layer forwarding. The research motivated by recurrent neural networks uses the learned *gating units* to control the rate of information flow which gives the benefit of individually better responding on each of different input data.

**Figure 3:**
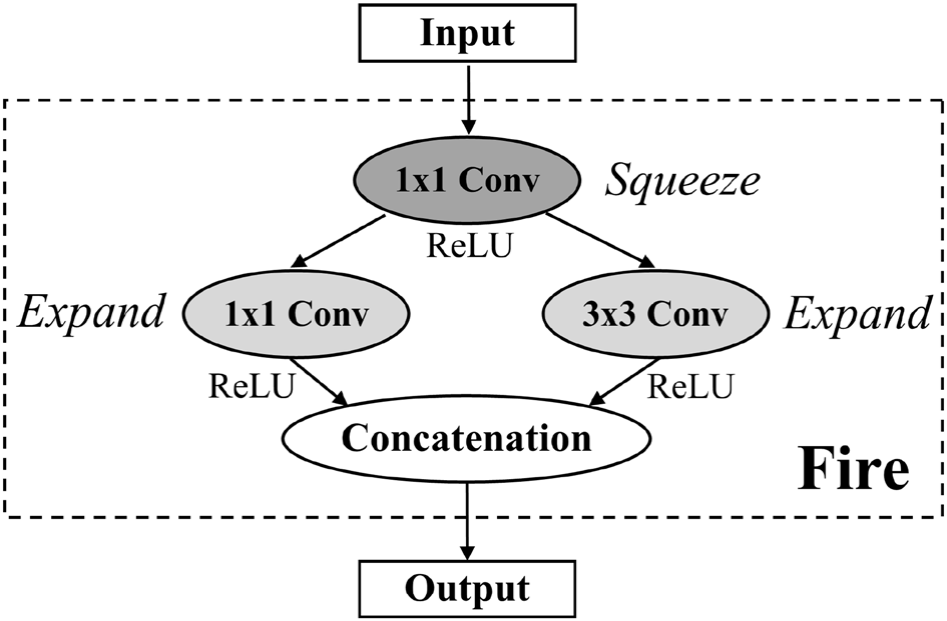
Fire module^1^.

*Fire module* SqueezeNet^1^ introduces *Fire module* shown in Fig. 3 which splits a regular convolution layer into two sub-layers called *squeeze* and *expand* layers. The squeeze layer demonstrates the usage of 1x1 convolution filters to decrease several input channels into each convolutional layer. The expand layer minimizes the number of model parameters while preserving a level of accuracy via a given ratio of using 1x1 and 3x3 convolution filters instead of using the whole of 3x3 filters in order to extract features from the input transformed by the squeeze layer. Both results are concatenated as an output of the module. According to the empirical results, SqueezeNet reveals a capability of prediction at the same level of accuracy as AlexNet (citeAlex whereas using 50x fewer parameters.

**Figure 4:**
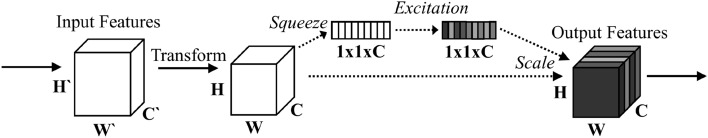
Squeeze and excitation operations in the SE block^11^.

*SE block* Squeeze-and-Excitation Network (SENet)^11^ proposed the *SE block* which can be attached to a convolutional layer as shown in Fig. 4. The SE block is used to investigate the importance and relationship of each feature of output channels. It applies global average pooling on each feature map to derive the channel descriptor (*squeeze operation*) which is then fed into two fully-connected layers to further learn the feature importance (*excitation operation*). Thus, the block has a role to rescale the original feature maps, strengthening the significant ones, and suppressing the less impact ones.

Using the attached SE blocks requires additional parameters compared to the original model. Our work exposes the worthiness of enhancing considerably the level of accuracy with the minimal additional cost of memory size and computation. In other words, the SE block attachment, rather than adding convolution layers, can help to improve accuracy on the deep model with a small increment of parameters.

### Related works

The concept of neural architecture search (NAS) has been established years ago. The goal is to automatically discover the optimal architecture for specific tasks. The area has recently become active in deep learning research. Various techniques have been applied to NAS and demonstrated great success on a variety of deep learning tasks, such as image recognition, natural language processing etc.^16^

In earlier years, reinforcement learning (RL)-based NAS methods have been used to search for the architecture. It has a controller to generate the potential neural networks being trained to acquire their performance as a reward for evolving the controller with a reinforcement learning algorithm. The earlier example is^17^ where the whole networks are searched. NASNet^18^, proposed a design of search space to discover only an architectural building block, instead of the entire network architecture, on a proxy dataset and then the learned block, i.e. scaling a number of the learned block, is transferred to the targeted dataset. It can save computational time and resources for searching and enable transferability to the related tasks.

ENAS^19^ improved the efficiency of searching by sharing weights of all possible architectures of the search space via creating a large computational graph gathering all possible models, and then every subgraph, i.e. any sampled architecture, utilizes their corresponding weights together instead of retraining the new sampled architecture from scratch.

While the algorithm of the NAS style performs an automatic search on the whole architecture, searching the micro-architecture types is also possible. For some classification tasks, the need to adjust the portion of the network can lead to better performance. Exhaustively searching for possible architectures consumes time resources and effort.

With the rise of AutoML framework, it becomes possible to implement the micro-architecture search easily. AutoKeras^20^ is one of the tools for AutoML which can automate the model finding. The tool relies on the NAS algorithm and it has three steps: Update, Generate, and Observation. In the update phase, it trains the Gaussian process model from existing architectures. and During the generation phase, it creates the training model based on the acquisition function (UCB)^21^. Also, several optimizations are considered to limit the search space such as editing distance and tree optimization. In the observation step, the generated model is trained and the accuracy is observed. Keras Tunner^22^ is a hyperparameter optimization framework for the hyperparamter search. It contains a built-in search algorithm such as Bayesian optimization. The algorithm has a behavior based on randomness, but its search time and results are acceptable. Furthermore, the framework also provides other recent search algorithms such as Hyperband^23^, and the traditional random search. The framework also allows the new implementation of the search algorithm.

In the recent work^24^, the Autopytorch framework utilized the multi-fidelity approach to optimize the hyperparameters^25^ The approach utilizes less cost and uses a meta-learning approach.

Squeeze-and-Excite block (SE block)^11^ is a kind of attention mechanism used previously in Seq2Seq networks. The concept is to concentrate on more useful feature channels than the less useful ones. Figure 4 focuses on the channel attention. Such a mechanism is increasingly popular among many researchers who study at the micro-architecture level.

To learn the channel importance of a given immediate result from any module of CNN, SE block used on SENet learns and performs feature re-calibration to highlight the informative feature channels. It aggregates the spatial information of each channel by using global average pooling (GAP) operation and then these channel representations are learned their importance through a bottleneck with two fully connected (FC) layers.

While SENet uses only GAP operation for embedding information of each channel, there are other variations. CBAM^26^ uses additional information via max-pooling operation. Global information embedding is utilized by the shared two FC layers to compute and combine both results, i.e. channel importance, using the element-wise summation. Not only the channel attention module but also CBAM presents a spatial attention module to refine the feature map along a spatial dimension.

The above-mentioned channel attention modules moderately increase the model complexity. Although the bottleneck with two FC layers proposed can reduce a fewer number of parameters when compared to the non-bottleneck version, it causes a channel dimensionality reduction during learning. ECA-Net^27^ selected 1D convolution operation instead of the two FC layers to perform local cross-channel interaction for calculating the channel importance. The local cross-channel interaction strategy helps avoid the problem of channel dimensionality reduction and still preserves the performance while significantly decreasing model complexity.

In this paper, we demonstrate the proper use of SE blocks: by exploring the block attachment positions and variations. The idea can be adapted to explore more options like in CBAM and ECA-Net.

## Design methodology

In this section, we explain how we add flexibility to the models to facilitate the exploration process. The variability is divided into two levels. The first is the machine learning parameters and the hyper-parameters of the layers. Both are handled directly by AutoKeras hyperparameter packages.

Secondly, at the micro-architecture level, the user may explore the possible use of e.g, residual blocks, the number of inserted blocks and the location of insertion, etc.

**Figure 5:**
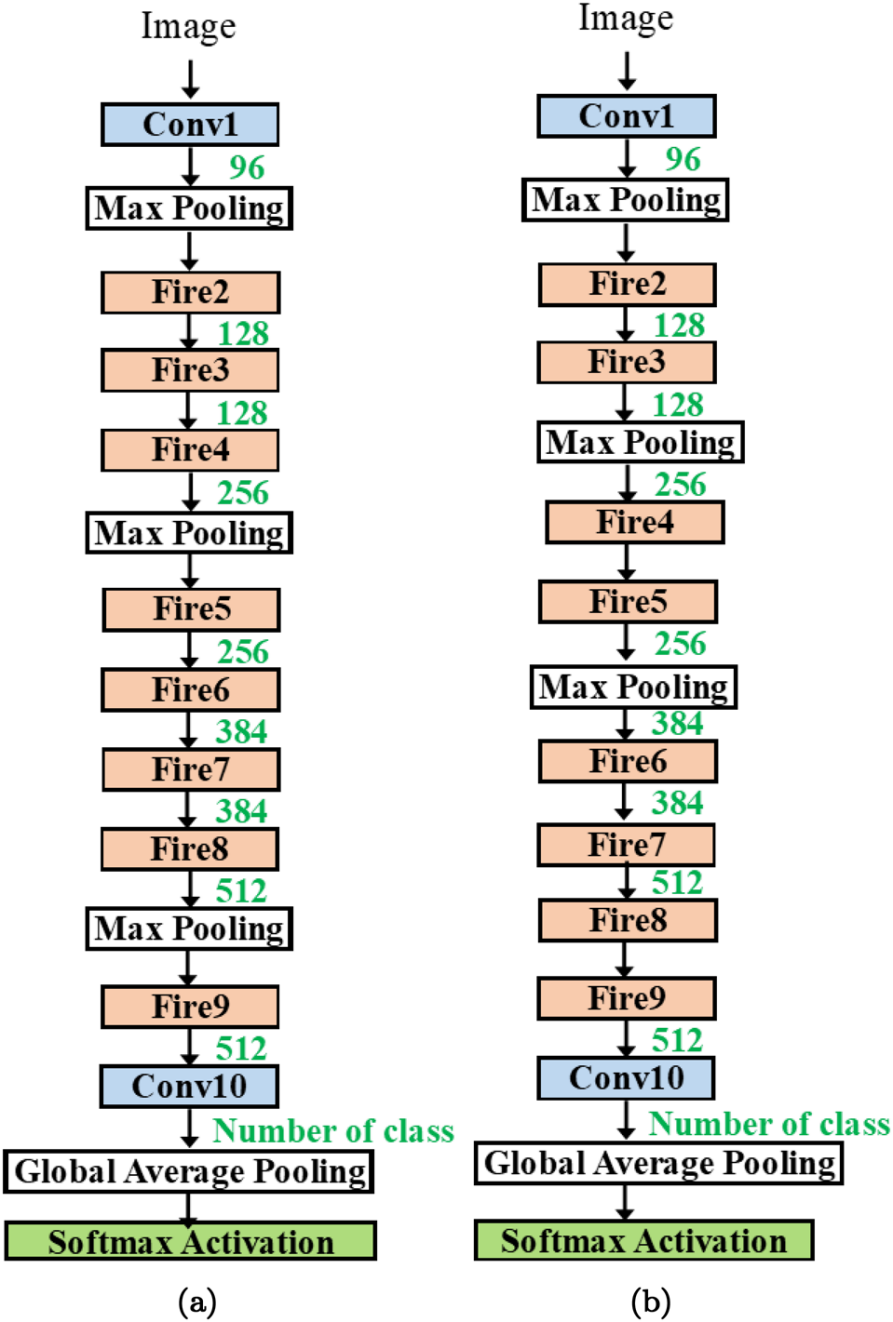
(**a**) SqueezeNet and (**b**) SqueezeNet V1.1. baselines.

### Baseline architecture

In Fig. 5, we present the baseline architecture used in the methodology. The SqueezeNet architecture consists of 7 fire modules (fire2-fire9). The fire module is depicted in Fig. 5. The input size is 224$\times$ 224. The implementation is adopted from^28^.

In Fig. 5a, the original SqueezeNet contains 3 fire modules followed by a max pooling layer, then 4 fire modules, and a max pooling layer again. The two final layers are the 9th fire module (fire9) and the 10th convolution (conv10) for classification (instead of the fully connected layer). In Fig. 5, max-pooling layers are inserted differently. In particular, it is inserted in every two modules. Both have (fire2–fire9) and 1 convolution layers.

In reality, the number of fire modules can be varied as demonstrated in the original work. Thus, we add on the first flexibility by introducing how to create a network with a flexible length.

**Figure 6:**
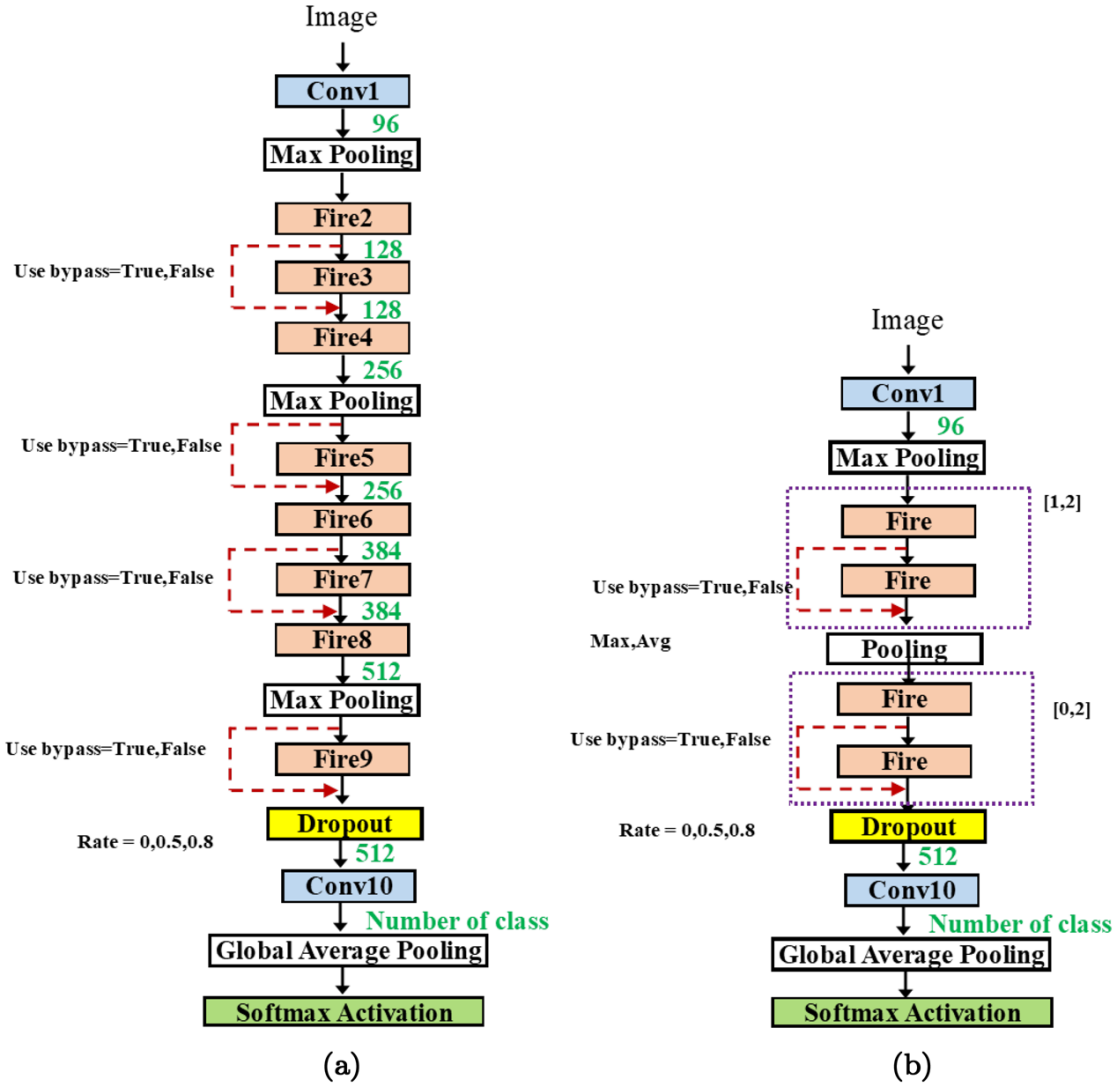
(**a**) SqueezeNet with auto-bypass (**b**) SqueezeNet with auto-length and more hyperparameters.

### Adding variable length and bypass

In the original paper of SqueezeNet, the authors proposed to have bypass connections. The bypass connections skip only the odd fire modules due to the compatibility of dimension sizes on the combined layer. Figure 6a shows the bypass configuration. In the first modification, we propose to put the flag on the bypass connection. In Fig. 6, the bypass connection is shown in a dashed line i.e., the connection can either be inserted or not inserted. After the last fire module, the dropout layer is added. It has a dropout rate as a hyperparameter whose possible values are in [ 0, 0.5, 0.8].

Since the fire modules are used in pair, we propose to use the flexible number of fire modules in pairs. Figure 6b shows the two dashed rectangles which highlight the groups of two fire modules. In each group, the second fire module can still be coupled with the skip connection. The first group has a flexible length, either 1 or 2 since it is required to have at least one fire module. In the second group, the possible number of fire modules is 0, 1, 2. This leads to the total possibility of up to 4 groups, or equivalently up to 8 fire modules. There are choices of pooling operations, either average or max pooling, as well.

**Figure 7:**
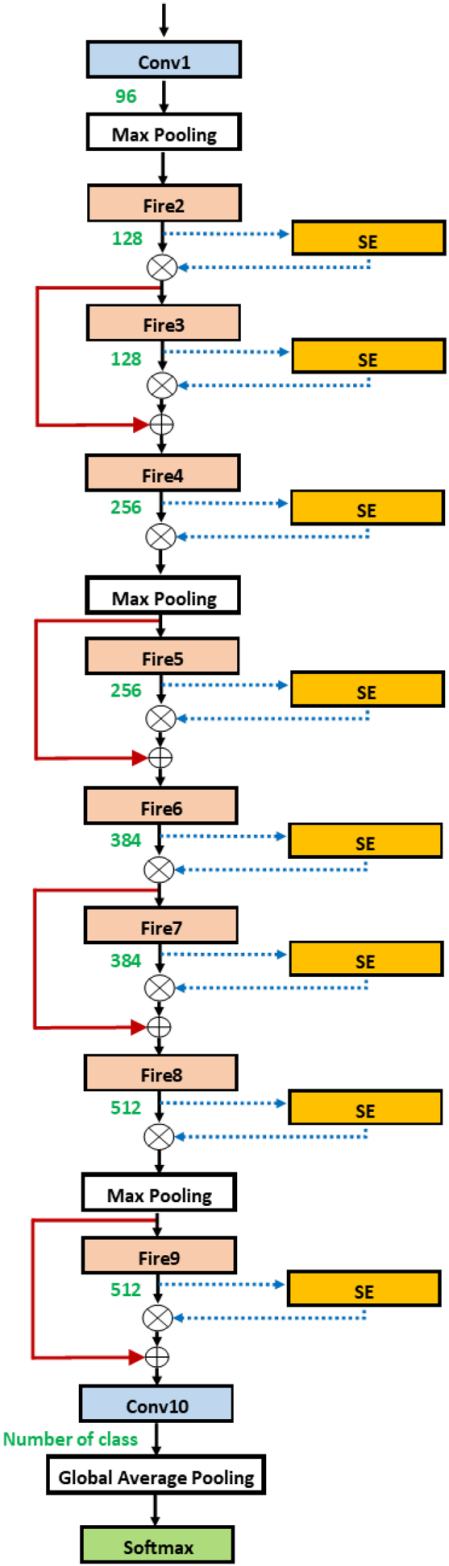
SqueezeNetSE^29^.

**Figure 8:**
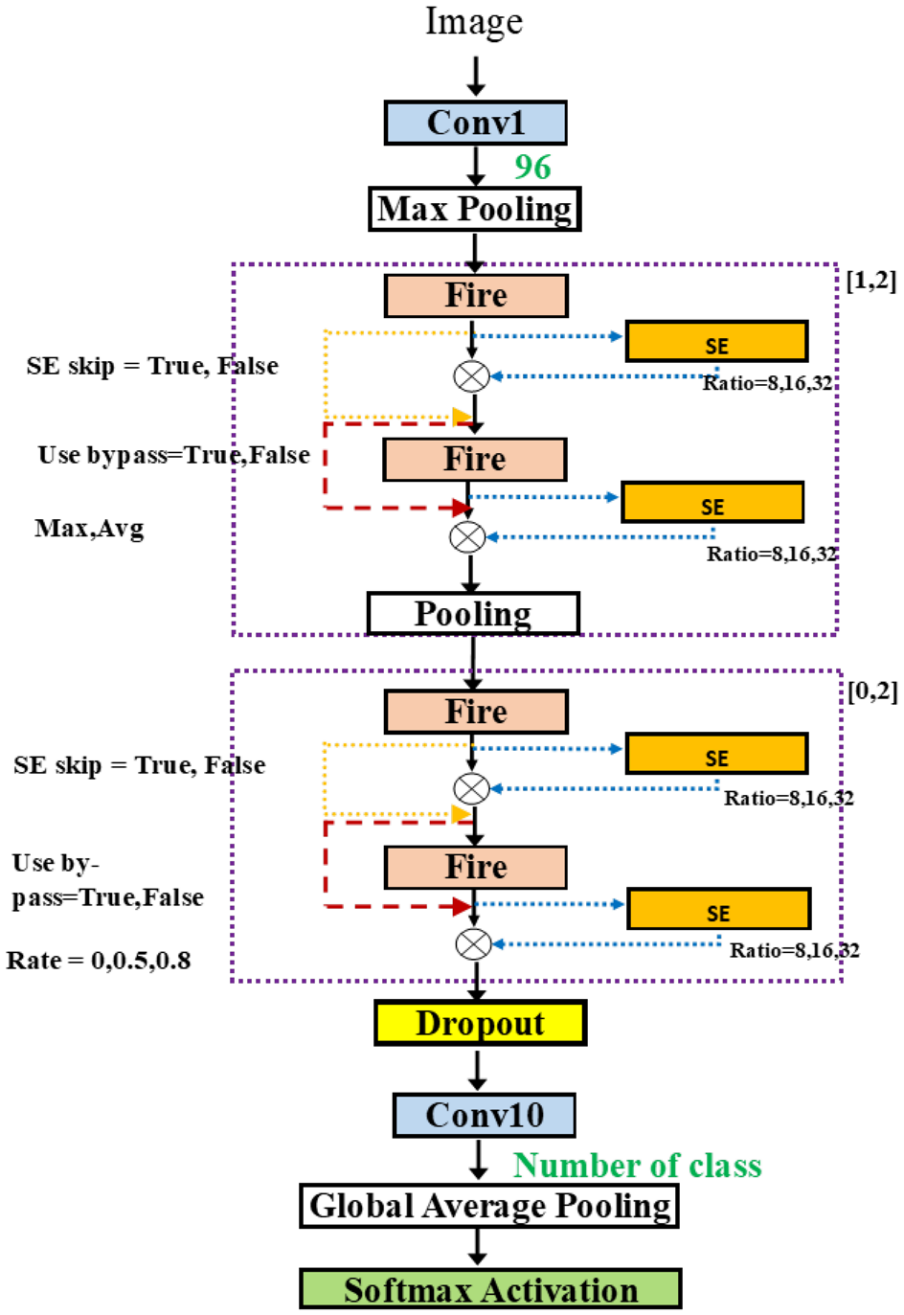
SqueezeNetSEAuto.

### Module insertions

Adding SE block may yield accuracy improvement^11^. It is a channel-based attention module and can be easily attached to the baseline network, in a similar way as the residual operation. However, there are many possible insertion points for a deep network. Considering one by one each is time-consuming^29^. Figure 7 presents SqueezeNet with SE block insertions. It is seen that there are many possible points of insertion after the fire modules.

In Fig. 8, we can attach the blocks in various positions in SqueezeNet. Adding more flexibility, we take the network from Fig. 6b and add the possible connection of SE block after each fire module. The group in the dashed rectangle now contains the SE insertion. The hyperparameter for SE block is the squeeze ratio valued in [ 8, 16, 32]. Also, the variable skip connection is added to, perhaps, bypass the SE block.

**Figure 9:**
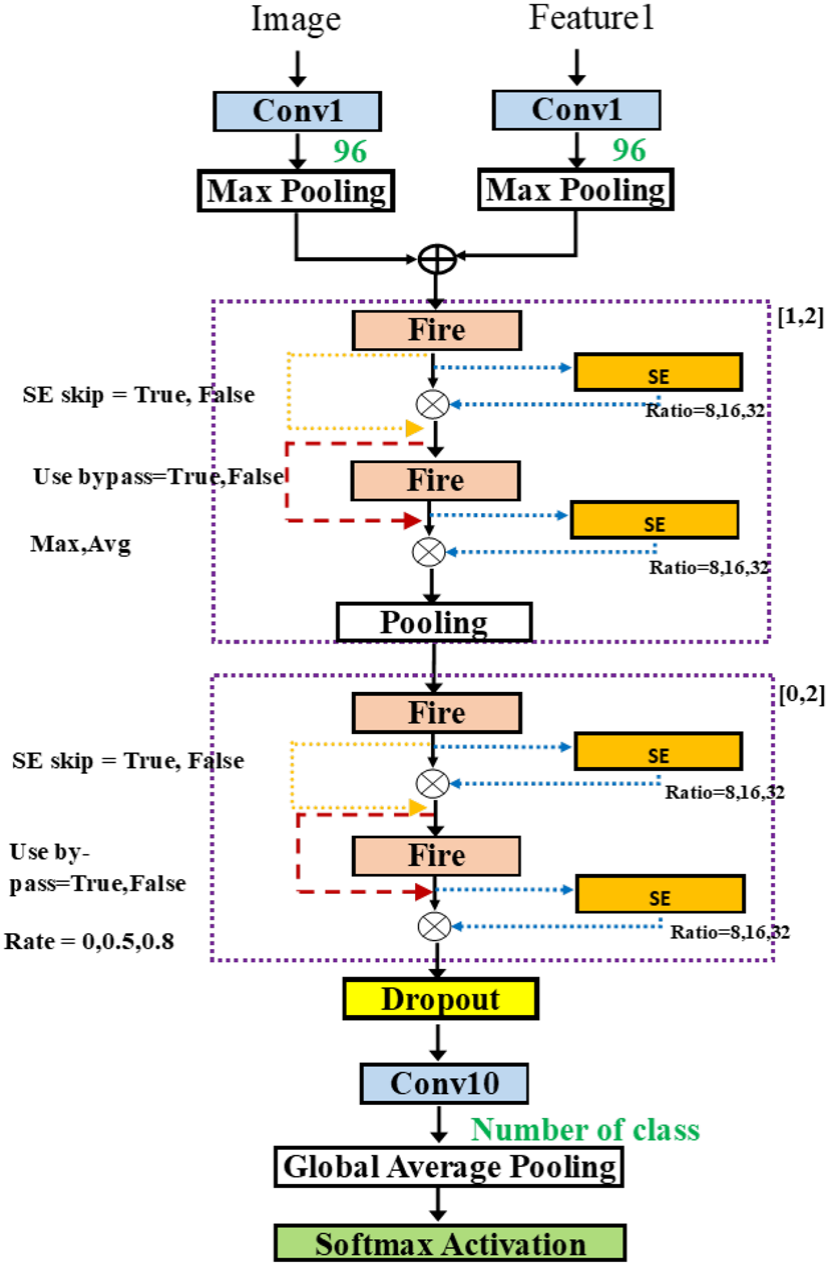
SqueezeNetSE1Auto.

**Figure 10:**
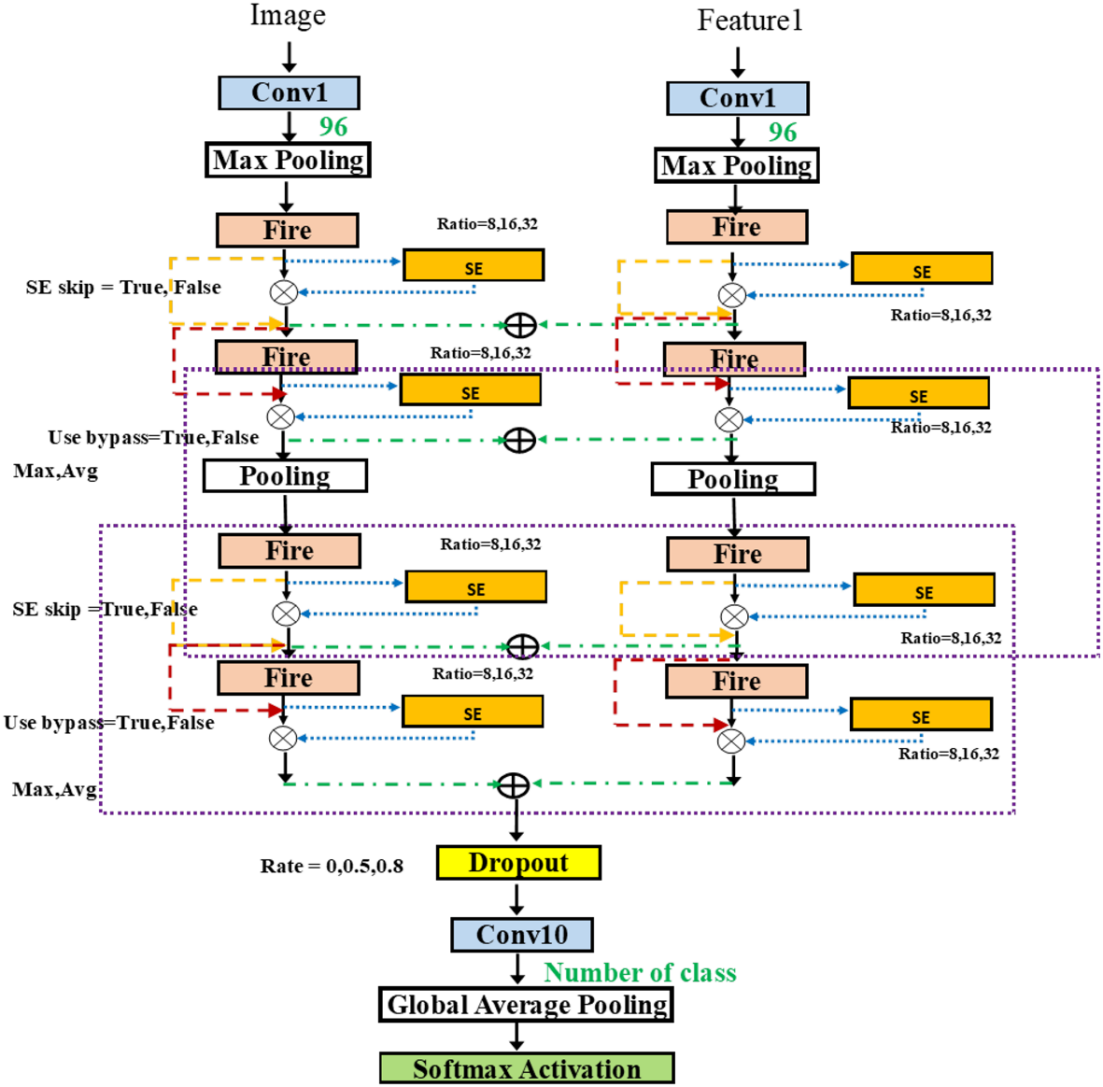
SqueezeNetAutoSEM.

### Multiple input merging

At last, due to the concept of the neural network, if some domain knowledge is added, the network can yield higher accuracy. Thus, adding input features can improve the accuracy. However, adding too many features can lead to over-fitting or high computation without the improvement of accuracy.

Figure 9 shows the typical approach to merge the two inputs, using the addition operation. In some networks, such as Siamese network^30^, the use of two models was proposed. Each input is fed into each model and then the merging of outputs is done at the last stage. In our methodology, we consider the addition of inputs at different layers. Thus, this implies the combining of multiple features at the flexible merging layer. Figure 9 is improved from Fig. 8 by merging after the first layer. Finally, Fig. 10 shows the variable merging points. Note that there exists only one merge point. After the two paths are merged, only one path remains.

**Figure 11:**
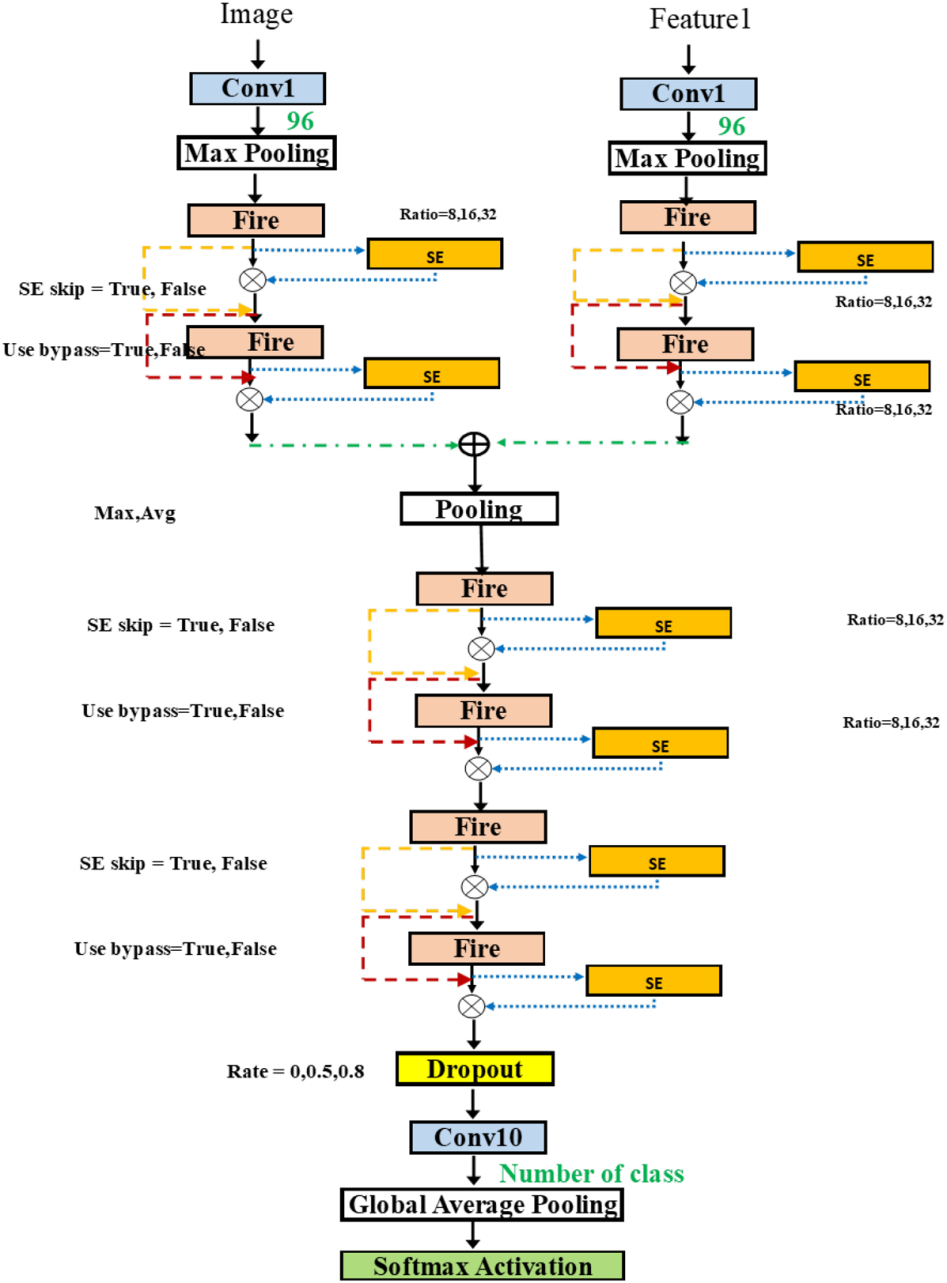
SqueezeNetSEMAuto (Example).

In Fig. 11, the selected architecture contains a first block of two fire modules, and then the two outputs are merged. After that, there are a pooling layer and two consecutive pairs of fire modules. For each layer, the hyperparameters are selected. The concept can be expanded to merge any number of inputs.

The proposed methodology presents the concept of adding varieties for architecture exploration in the three aspects. In the next section, we conduct experiments using the methodology to find the model architectures with high accuracy. The framework will facilitate the model architecture exploration process.

## Experiments

The experiments compare the results of 4 types of micro-architecture explorations based on the previous section.

1. The variable number of fire modules,
2. The addition of skip connections,
3. The insertion of the SE blocks in various places, and
4. The multiple-input merge points.

The two data sets: CIFAR-10^31^ and Tsinghua Facial Expression datasets^32^ is used for the experiments. Three search strategies are executed to perform model searching and the validation accuracy is reported. We measure the top-10 model sizes of the best solutions found. Some of the found solutions yield better accuracy than the baselines with the smaller model size.

We divide the section as follows. First, we report the results on CIFAR-10 benchmark. The goal is to find a suitable architecture by varying the micro-architecture connection and hyperparameters. Second, the results for the recent facial expression dataset are reported. For this data set, we also extract the landmark feature from the dataset. The feature is combined with the default inputs where the merging locations are explored. The main goal is to find out, whether adding input features yield a model with better accuracy. This demonstrates the need for combining inputs to the model with possible merging layers.

### Hypothesis

In the experiments, the assumptions have been set up for training. Table 1 presents the machine learning and hyperparameters.

**Table 1:** Machine learning and Hyper parameters.

Parameters	Possible values
Learning rate	0.001, 0.0001
Optimizer	RMSprop, SGD
Pooling	Average, Max
Dropout	[0, 0.8] (default=0.4)
Squeeze ratio	8,16,32

From Table 1, the variable learning rates are 0.001 and 0.0001. The two optimizations are considered: SGD, and RMSprop. We fix the batch size to 32. The variable hyperparameters are assumed for the dropout layer. The dropout rate is ranging between: 0 and 0.8 and the default value is 0.4. For the pooling layers, there are two choices of operations: max pooling and average pooling.

For SqueezeNet, there is another parameter, compression ratio. We set the compression ratio to 1. For SE block, the squeeze ratio parameter we choose is among values: 8, 16 and 32. The maximum epoch is 100. The early stopped method is used if the accuracy is changed for more than 5 iterations.

The three search algorithms are tested: Random, Hyperband, and Bayesian from Keras Tuner^22^. For Hyperband, the algorithm takes the maximum epoch instead. We set it to 100. For the Bayesian search algorithm, the alpha value is 0.001, the beta is 2.6, the number of initial points is 2 and the number of trials is 100. For Random and Bayesian, the number of trials is also 100. With these search schemes, the parameters are sampled and the model is built with such parameters. Then, the input dataset is fed in a batch for training. The training is done for the whole number of epochs and then validation is performed. If resources are permitted, the large scaled dataset can be used since it will be split into batches and utilize distributed training which Keras Tuner also supports.

### CIFAR-10 dataset

For CIFAR-10, the total data set is 60,000 and each image size is 32$\times$32. It has 10 classes with 1000 images per class. The data set is pre-divided into 50,000 training images and 10,000 testing images.

Table 2 shows the accuracy of each model for the three search algorithms. The parameters in Table 1 are used. The first two models are SqueezeNet and SqueezeNet11 presented in Section “Baseline architecture”. Then, SqueezeNetAuto is the model in Section “Adding variable length and bypass”. SqueezeNetSEAuto is the model in Section “Module insertions”. We vary the sample size of the data set for training and testing to see the effect on the accuracy. The sampling sizes are 10%, 40%, and 100%. The purpose is to see how the sampling size affects the maximum accuracy obtained while minimizing the training time. SqueezeNetSEAuto-0.1 is SqueezeNetSEAuto trained with only 10% of the training and testing dataset. Similarly, SqueezeNetSEAuto-0.4 and SqueezeNetSEAuto-1 utilize 40% and 100% of the whole data set respectively. The searching strategy does not affect the solutions much. Random search performs well enough compared to Baysian and Hyperband. It runs a little faster than the two. We use the early stopped approach. Then, all of the experiments may not train until the maximum number of epochs.

**Table 2:** Accuracy results for CIFAR-10.

Models	Random	Hyperband	Baysian
SqueezeNet	0.507	0.235	0.52
SqueezeNet11	0.538	0.264	0.556
SqueezeNetAuto	0.556	0.579	0.592
SqueezeNetSEAuto-0.1	0.572	0.570	0.558
SqueezeNetSEAuto-0.4	0.697	0.706	0.693
SqueezeNetSEAuto-1	0.760	**0.763**	0.746

Significant values are in [bold].

From Table 2, using SqueezeNetAuto mostly yields models with better accuracy compared to the baseline. When adding SE blocks, the accuracy is improved further. The model architectures are reported in Table 3 for SqueezeNetAuto for the three search algorithms.

In a row, “# fire module blocks in (1)” presents the number of repeated blocks used in the first dashed block in Fig. 8 while “# fire module blocks in (2)” is the number of repeated blocks of the second one. Each dashed block contains two fire modules. Row “use bypass”, shows whether the skip connection in the red dashed line in Fig. 8 is needed.

The results show that the selected three models have similar architecture. In the table, only 1 fire module block is needed for each part of the three algorithms. Thus, all models use only fire2, fire3, fire4, fire5. The small difference is the use of a bypass for each section. For example, Random and Bayesian algorithms select no bypass connection for fire3 and Hyperband algorithm selects a bypass connection for fire3. For fire5, Random and Bayesian algorithms select the bypass connection while Hyperband algorithm does not pick the bypass connection. All use average pooling after fire3 and use max pooling after fire5. Random and Bayesian algorithms select the SGD optimizer while RMSprop is chosen for the optimizer. Thus, the size of the three is about the same, compared to the baseline where all fire2-fire9 are used, to achieve similar accuracy.

**Table 3:** Models obtained by various searches.

Parameters	Random	Hyperband	Bayesian
Learning rate	0.001	0.001	0.001
# fire module blocks (1)	1	1	1
Use bypass (1)	FALSE	TRUE	FALSE
Pooling	avg	avg	avg
# fire module blocks (2)	1	1	1
Use bypass (2)	TRUE	FALSE	TRUE
Dropout rate	0.48	0.43	0.48
Optimizer	SGD	RMSprop	SGD
Accuracy	0.556	0.578	0.592

Consider the solution obtained by SqueezeNetSEAuto-0.4 in Table 4. Row “use SE” means whether the SE block (in yellow) in Fig. 8 is needed. Note that the dashed block of fire modules has two SE blocks inside. Each SE block may have a skip connection inside. This is shown in Boolean in row “SE Skip”.

All the selected models contain the same number of fire modules. In Table 4, the selected models from Random and Hyperband algorithms, only 1 block is needed for each part and from Bayesian algorithm two blocks are selected for the first part and zero block is used for the second part. Thus, totally 4 fires modules: fire2, fire3, fire4, fire5 are used. Random selects 2 SE blocks at fire2, fire3, fire4, and fire5.

**Table 4:** SqueezeNetSEAuto (0.4) for three algorithms.

Parameters	Random	Hyperband	Bayesian
Learning rate	0.001	0.001	0.001
# fire module blocks (1)	1	1	2
Use bypass (1)	FALSE	FALSE	FALSE,FALSE
Use SE	TRUE,FALSE	FALSE,FALSE	TRUE,FALSE, FALSE,TRUE
SE Skip	TRUE,TRUE	FALSE,TRUE	TRUE,FALSE, FALSE,TRUE
Squeeze ratio (1)	16	32	32,8
Pooling	avg	avg	max,avg
# fire module blocks (2)	1	1	0
Use bypass (2)	FALSE	TRUE	–
Use SE (2)	TRUE,TRUE	TRUE,FALSE	–
SE Skip (2)	TRUE,TRUE	TRUE, TRUE	–
Squeeze ratio (2)	32	32	–
Dropout rate	0.16	0.67	0.49
optimizer	SGD	RMSProp	SGD
Accuracy	0.697	0.705	0.693

**Figure 12:**
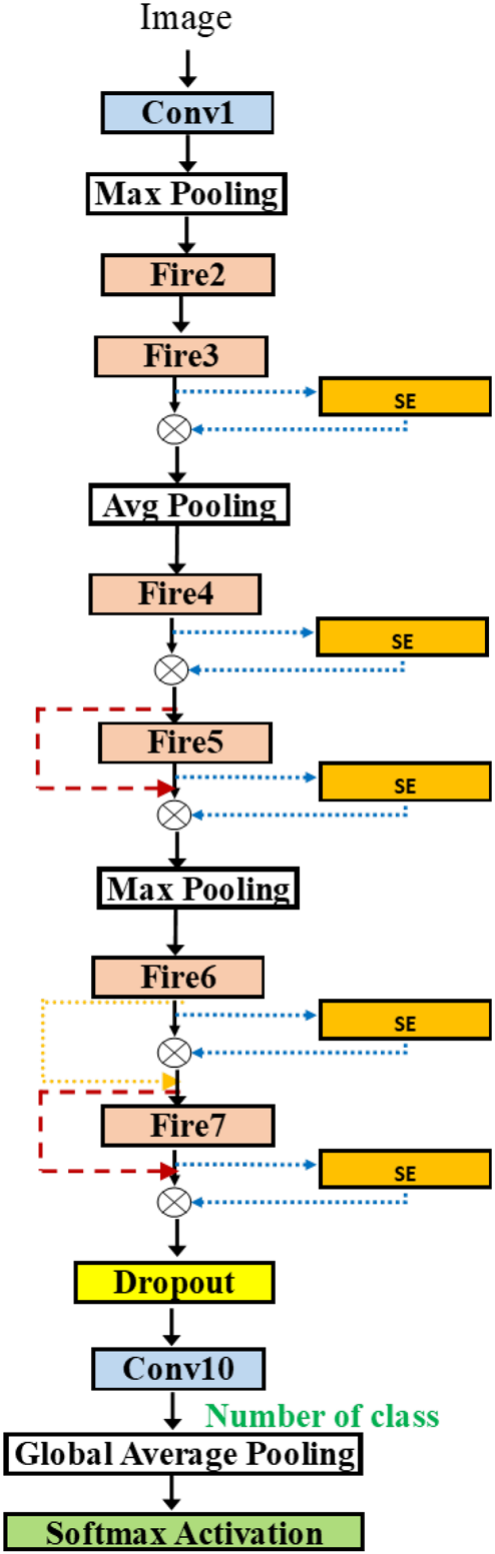
Model from SqueezeNetAuto (Random).

**Figure 13:**
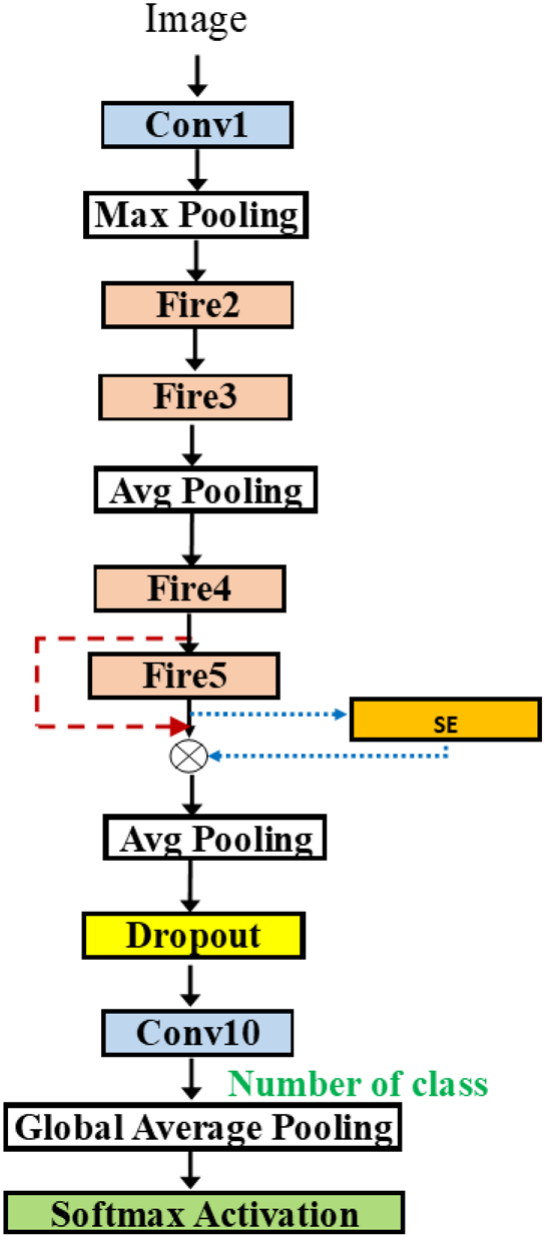
Model from SqueezeNetAuto (Hyperband).

**Figure 14:**
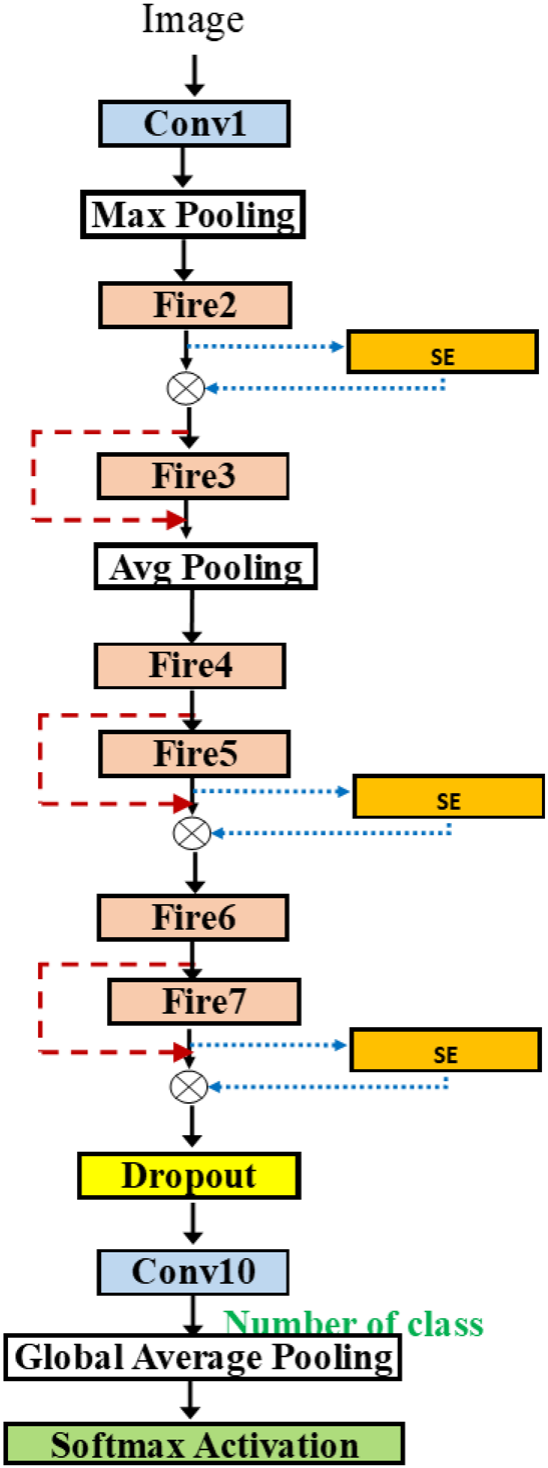
Model from SqueezeNetAuto (Bayes).

Table 5 shows the results when using all data to train the model. For Random and Bayesian algorithms, the selected models contain the three fire blocks while, for Hyperband algorithm, the model contains the two fire blocks. The number of SE blocks used is 5 inserted at fire3–fire7 for the Random algorithm (Fig. 12), 1 at fire5 for the Hyperband algorithm (Fig. 13) and 3 at fire2, fire5 and fire7 for Bayesian algorithm (Fig. 14) respectively. Thus, the Hyperband algorithm selects better models than others since it uses only 4 fire modules and 1 SE block while achieving the accuracy the same as the model obtained by the Random algorithm. The results imply that we can achieve a less complicated model with the same accuracy level.

**Table 5:** SqueezeNetSEAuto (1.0) for three algorithms.

Parameters	Random	Hyperband	Bayesian
Learning rate	0.0001	0.0001	0.001
# fire module blocks (1)	2	2	1
Use bypass (1)	FALSE,TRUE	FALSE,TRUE	TRUE
Use SE	FALSE, TRUE, TRUE,TRUE	FALSE, FALSE,FALSE, TRUE	TRUE,FALSE
SE Skip	–,FALSE, FALSE,FALSE	FALSE,FALSE, –,TRUE	TRUE,–
Squeeze ratio (1)	32,8	32,16	8
Pooling	avg,max	avg,avg	avg
# fire module in blocks (2)	1	0	2
Use bypass (2)	TRUE	–	TRUE, TRUE
Use SE	TRUE, TRUE	–	FALSE, TRUE, FALSE, TRUE
SE skip	TRUE, FALSE	–	–, FALSE, –, FALSE
Squeeze ratio (1)	32	–	32,32
Dropout rate	0.12	0.31	0.57
Optimizer	RMSProp	RMSProp	SGD
Accuracy	0.76	0.76	0.74

### Tsinghua facial expressions

**Figure 15:**
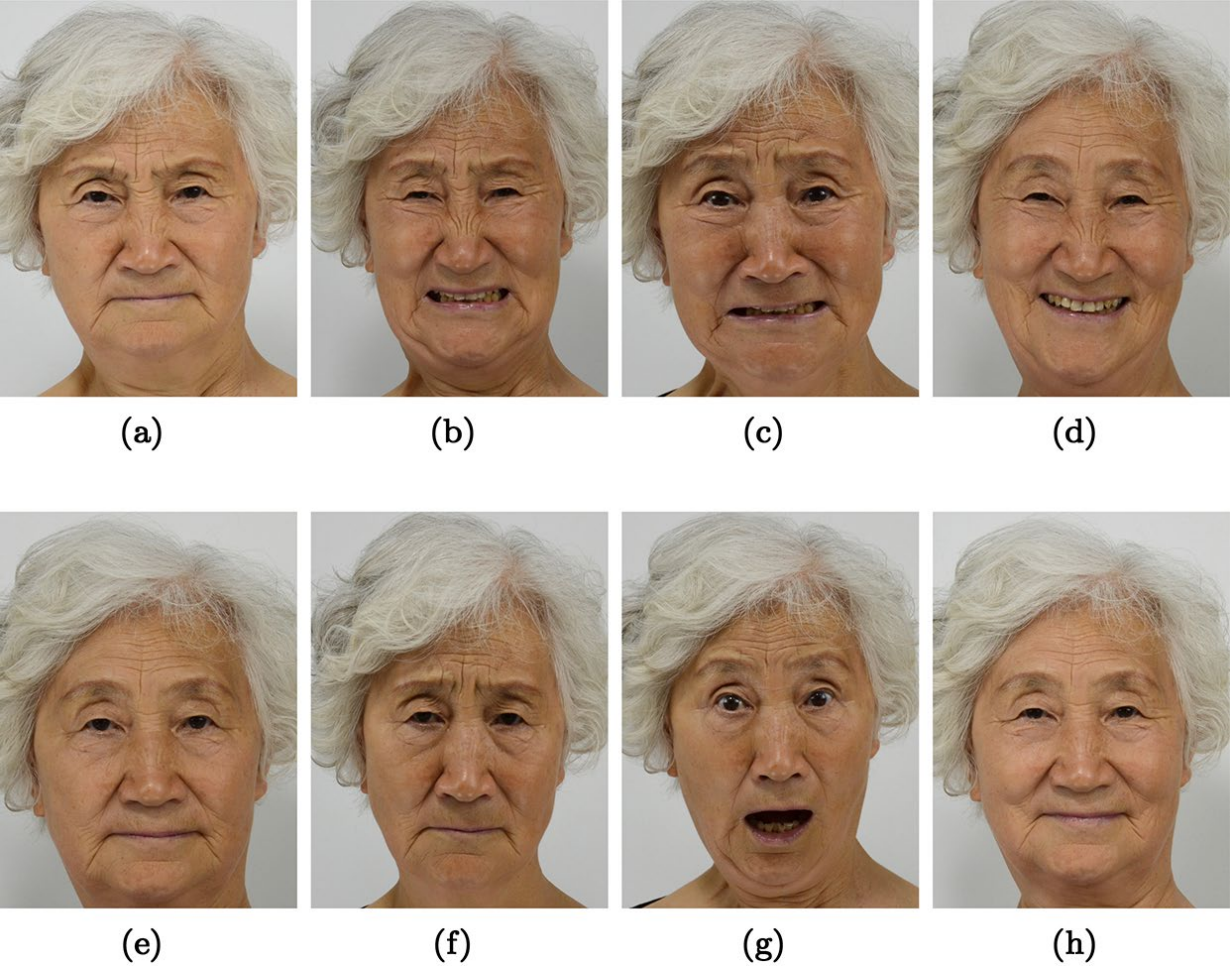
(**a**) Anger (**b**) Disgust (**c**) Fear (**d**) Happy (**e**) Neutral (**f**) Sad (**g**) Surprise (**h**) Content.

The data set contains 110 subjects, and each subject has 8 classes of expressions: sad, neutral, surprise, anger, disgust, happy, content. Each image size is 2,000$\times$1,500. The data set is publicly available at^32^. The example images are shown in Fig. 15. The data set is divided into 80:20 for training and testing sets respectively.

To utilize SqueezeNet, the input image is resized to 224x224. We first experiment on model searching. Table 6 reports the numerical results. This data set size is not as large as CIFAR-10 while the image size is large. We have to reduce the size to speed up the time to feed the input to the network.

**Figure 16:**
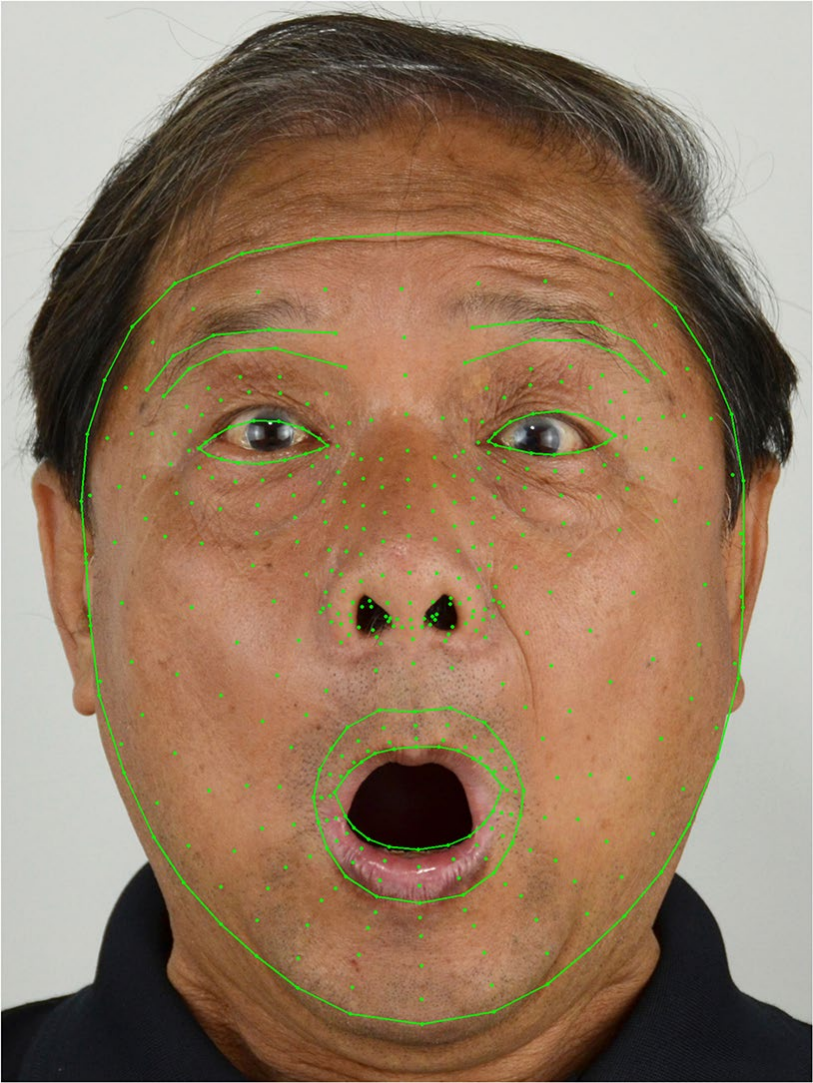
Facial landmarks extracted.

The auxiliary feature used is the 3D landmarks extracted by Mediapipe^33^. The face detector is based on BlazeFace^34^. The landmarks contain 468 points as shown in Fig. 16 Each point has coordinates as *x*, *y*, *z*. *x*,*y* values are normalized to [0,1] with respect to the width and height of the image respectively. The *z* is the landmark depth reference with the depth of the head center treated as the origin. The small value means the position is close to the camera. *z* uses the same scale as *x*.

**Figure 17:**
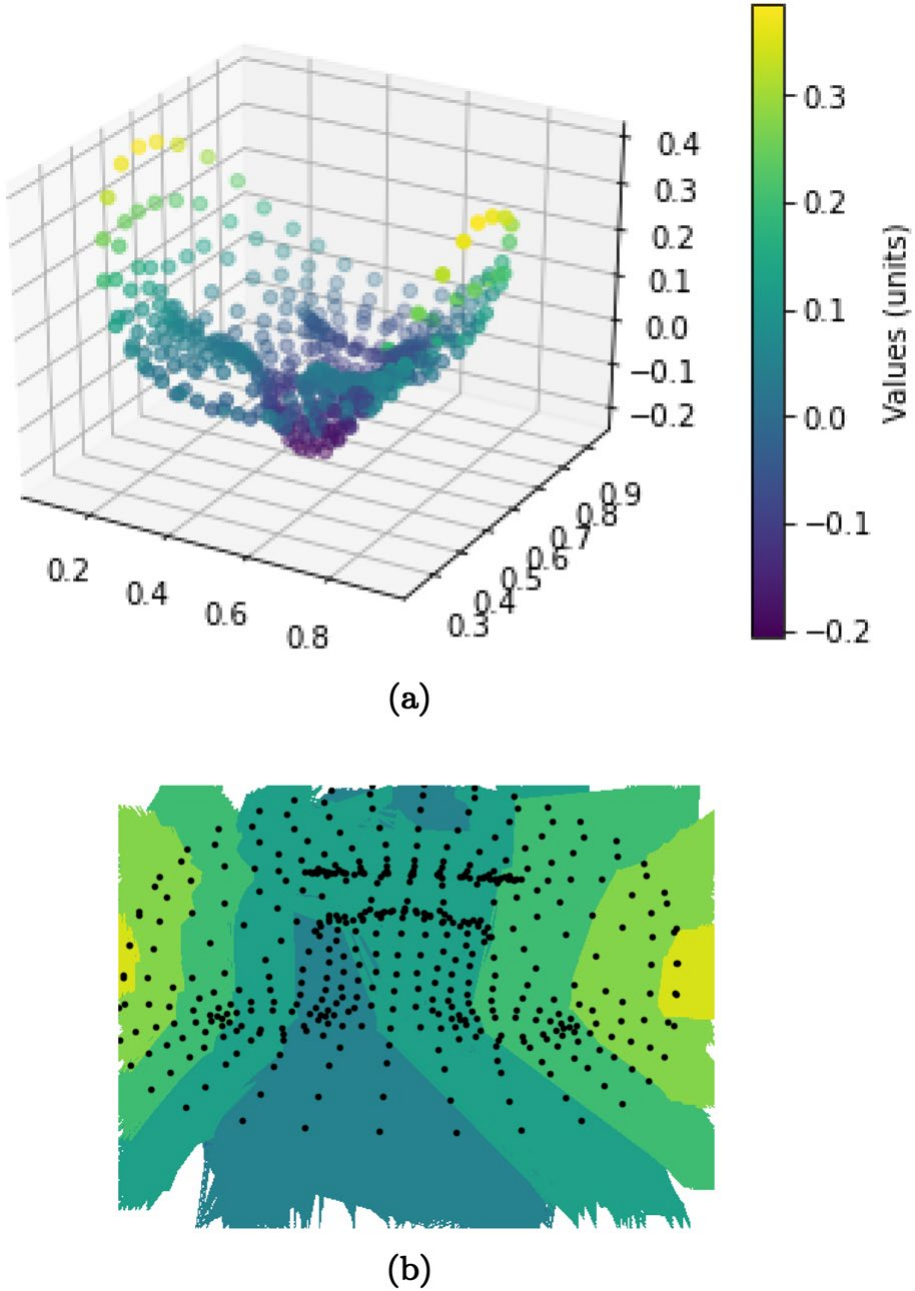
(**a**) Landmarks in 3D space (**b**) Depth interpolation.

Figure 17a presents the landmarks plotted in the 3D space. From the figure, we draw a depth map image in Fig. 17b. This is used as another input for SqueezeNetSEM experiments.

**Table 6:** Accuracy results for Tsinghua facial expression data set.

Model	Random	Hyper	Baysian
SqueezeNet	0.137	0.125	0.114
SqueezeNet11	0.125	0.114	0.137
MLPAuto	0.165	0.2	0.154
SqueezeNetAuto	0.159	0.182	0.314
SqueezeNetSEAuto	0.622	0.502	0.674
SqueezeNetSE1Auto	0.697	0.691	0.617
SqueezeNetSEMAuto	0.691	0.708	**0.714**

Significant values are in [bold].

Table 6 presents the validation accuracy for the baseline models and for the models proposed by our algorithm. In the table, SqueezeNet and SqueezeNet11 are the same one as in Table 3 where the hyperparameters are selected from Table 1.

MLPAuto is a multi-layer perceptron. We use the 468 landmark points of *x*, *y*, *z* from Mediapipe to create the classic MLP model. The number of dense layers is varied from [4, 10] and the number of hidden nodes is selected among [16, 32, $\ldots$, 512]. At the last two rows, SqueezeNetSE1Auto takes two inputs: image input and depth image input and merges them using the add operation after the first convolution and pooling layers. SqueezeNetSEMAuto takes the same two inputs but the merge point is also a parameter as in Fig. 10.

For the first three networks, the low accuracy is achieved through the varied hyperparameter. Thus, such a fixed structure obstacles the search exploration. When augmented with SE blocks, the accuracy can be significantly improved. This is the benefit by the SE block with the channel normalization.

Next, the question is how to insert the SE blocks and how many blocks are needed. We explore the SE block insertion by the algorithm SqueezeNetSEAuto presented in Section “Module insertions”. The algorithm leads to the superior architecture found by Random, Hyperband, and Bayesian algorithms respectively.

Tables 7, 8, 9 and 10 show the model sizes (in bytes) obtained from the three algorithms. Table 7 shows the model size in bytes of the top-5 models from SqueezeNetAuto. It is seen that the model size is quite small and the accuracy is low. Table 8 shows the model size in bytes of the top-5 model of SqueezeNetSEAuto. For the Random algorithm, it is seen that the model size is three times larger than that of SqueezeNetAuto, while the accuracy is about 4 times higher and similar to the solutions from the Hyperband algorithm. However, it is interesting that the solution offered by the Bayesian algorithm has the highest accuracy (0.674) while the model size (145,032) is a little higher than that of SqueezeNetAuto (123,880). This model architecture is depicted in Fig. 19.

**Table 7:** Model size and accuracy for SqueezeNetAuto.

Rank	SqueezeNetAuto					
	Random		Hyperband		Baysian	
	Model size	Acc	Model size	Acc	Model size	Acc
1	123,880	0.159	28,072	0.182	28,072	0.314
2	28,072	0.154	28,072	0.177	28,072	0.154
3	123,880	0.142	123,880	0.159	28,072	0.142
4	123,880	0.137	123,880	0.154	28,072	0.142
5	28,072	0.137	28,072	0.154	28,072	0.137

Table 9 presents the model size in bytes of the top 5 models obtained by SqueezeNetSE1Auto. At the first rank, Random algorithm yields the model that has higher accuracy (0.691) with the model size, 182,936 compared to the model from SqueezeNetAuto (123,880).

**Table 8:** Model size and accuracy for SqueezeNetSEAuto.

Rank	SqueezeNetSEAuto					
	Random		Hyperband		Baysian	
	Model size	Acc	Model size	Acc	Model size	Acc
1	362,208	0.622	381,940	0.502	145,032	0.674
2	473,316	0.605	985,288	0.417	175,604	0.605
3	432,552	0.565	1,085,028	0.394	145,032	0.571
4	171,052	0.531	1,085,028	0.382	145,032	0.560
5	388,244	0.502	1,085,028	0.360	145,032	0.537

**Table 9:** Model size and accuracy for SqueezeNetSE1Auto.

Rank	SqueezeNetSE1Auto					
	Random		Hyperband		Baysian	
	Model size	Acc	Model size	Acc	Model size	Acc
1	143,608	0.691	487,168	0.691	402,064	0.617
2	787,336	0.634	1,156,272	0.622	402,064	0.577
3	160,152	0.634	882,020	0.542	410,376	0.571
4	383,864	0.628	1,156,272	0.479	410,376	0.565
5	148,860	0.605	416,832	0.400	410,376	0.560

In Table 10, there is a solution found by Random algorithm achieving accuracy 0.691 with the model size 182,936. The highest accuracy, 0.714, is obtained by the model selected by Bayesian algorithm. The model size is about 7 times larger (884,688) that that is obtained by SqueezeNetAuto (123,880).

**Table 10:** Model size and accuracy for SqueezeNetSEMAuto.

Rank	SqueezeNetSEMAuto					
	Random		Hyperband		Baysian	
	Model size	Acc	Model size	Acc	Model size	Acc
1	182,936	0.691	445,664	0.708	884,688	0.714
2	453,568	0.685	176,664	0.668	884,688	0.714
3	280,552	0.611	176,920	0.577	894,300	0.708
4	429,376	0.605	573,792	0.548	884,688	0.702
5	1,159,592	0.605	505,656	0.502	884,688	0.697

**Figure 18:**
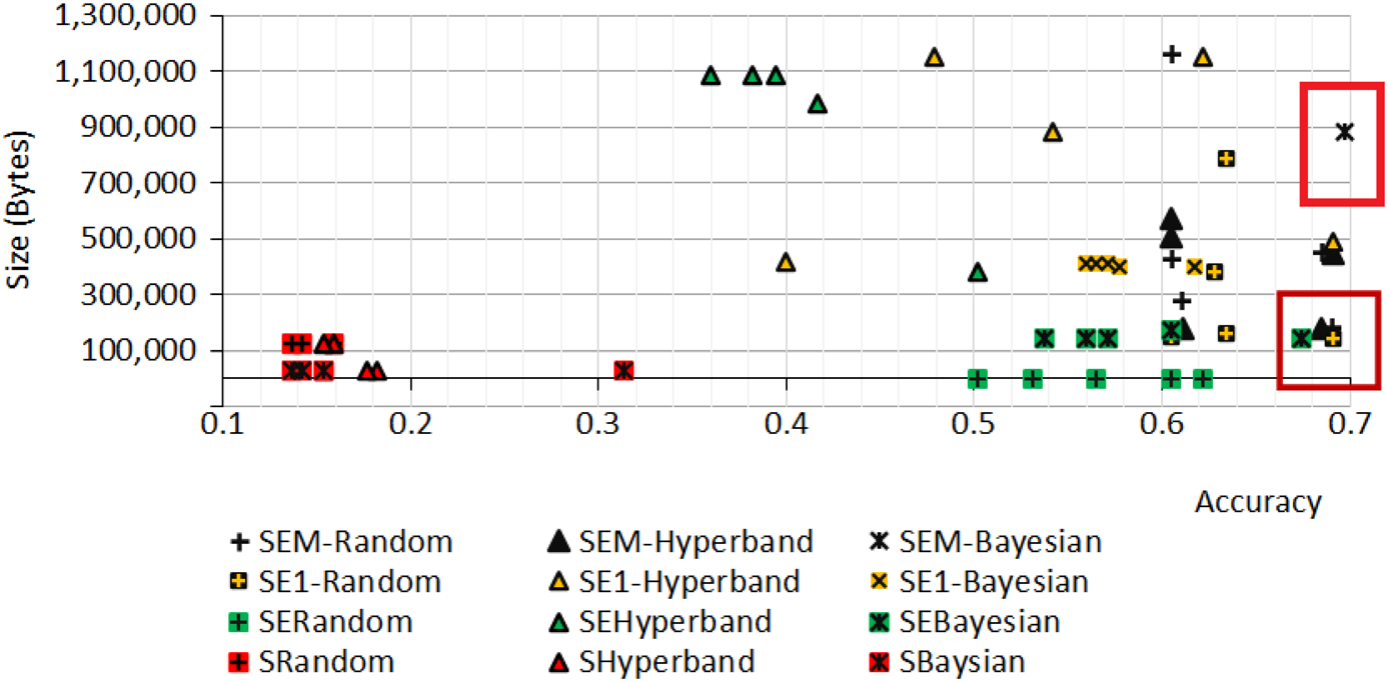
Comparing model sizes and accuracy for all tests.

**Figure 19:**
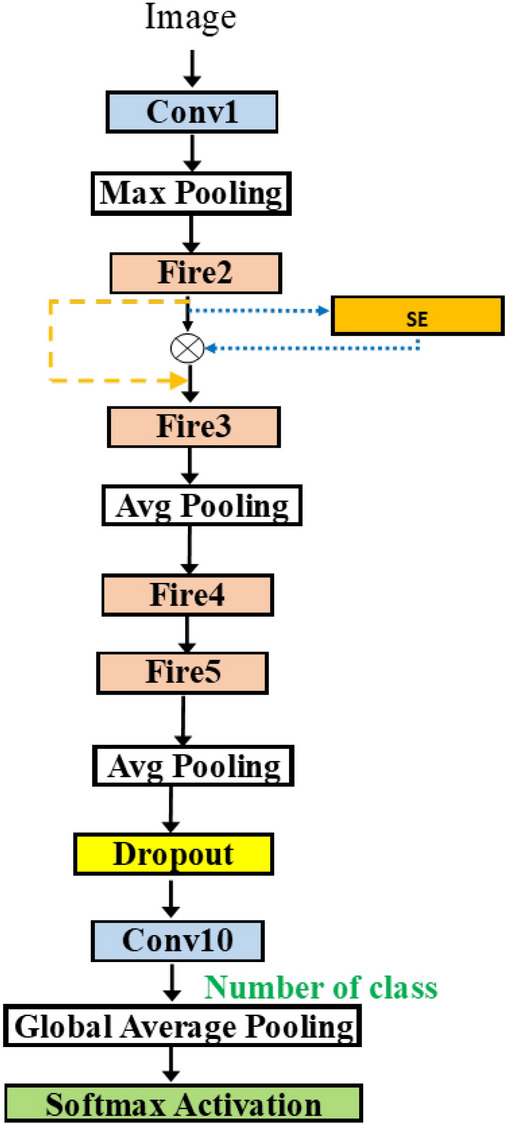
Model obtained by SqueezeNetSEAuto for Tsinghua data set (Bayesian), acc=0.67, size=14M.

**Figure 20:**
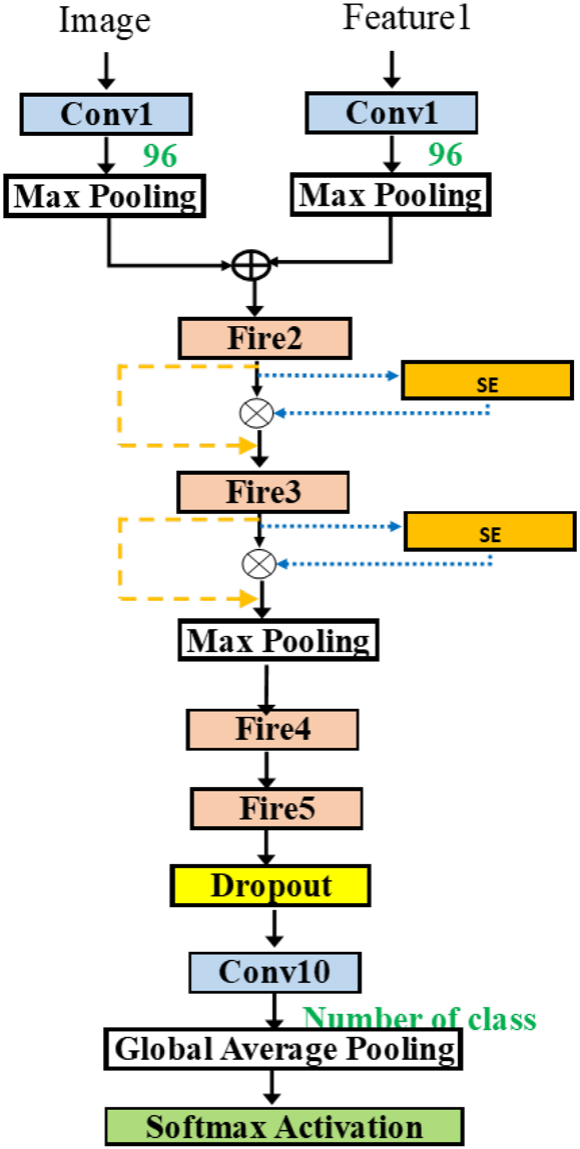
Model obtained by SqueezeNetSE1Auto for Tsinghua data set (Random), acc=0.69, size=14M.

**Figure 21:**
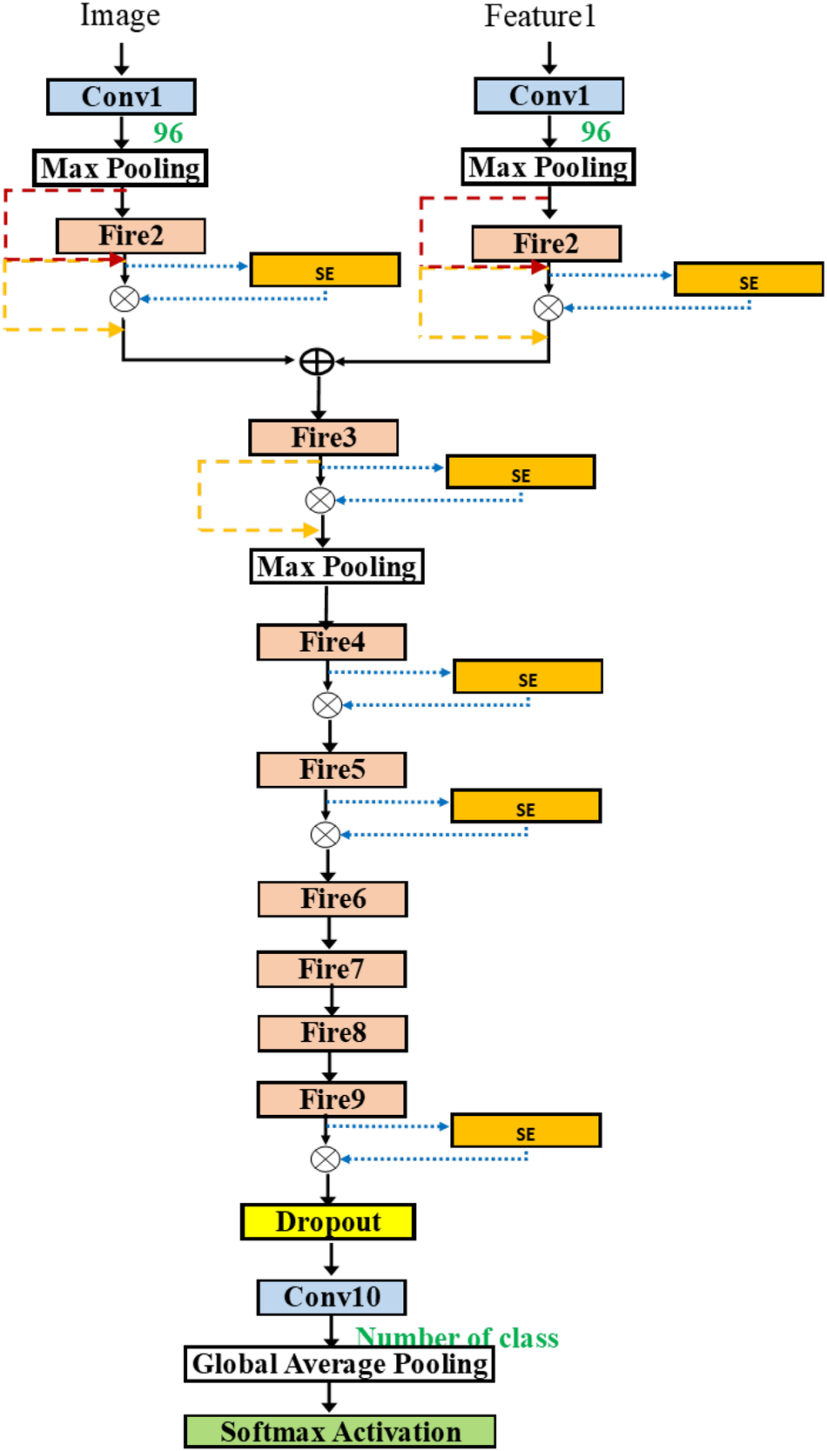
Model obtained by SqueezeNetSEMAuto for Tsinghua data set (Bayesian), acc=0.71, size=88M.

Figure 18 shows the comparison for sizes and accuracy among all cases in Tables 7, 8, 9 and 10. The highlight boxes show the best accuracy cases for the small model size. The cases are derived from SqueezeNetSEAuto (Bayesian), SqueezeNetSE1Auto (Random), SqueezeNetSEMAuto (Hyperband), and SqueezeNetSEMAuto (Random). Figures 19, 20 and 21 present the selected top three models from Tables 8, 9 and 10.

The experiments demonstrate that the proposed framework yields the exploration of models effectively. It can help the designer explore the possible micro-architectures at the same time as exploring the hyperparameters of the layers. In the future, we will customize the search algorithm suitable for the specific micro-architecture types.

## Other applications

The proposed approach can be applied to explore the micro-architecture change in the baseline architecture that requires similar tuning. In this section, we demonstrate the application to other tasks such as image segmentation and object detection tasks with the backbone architectures that have a similar micro-architecture style.

### Image segmentation

**Figure 22:**
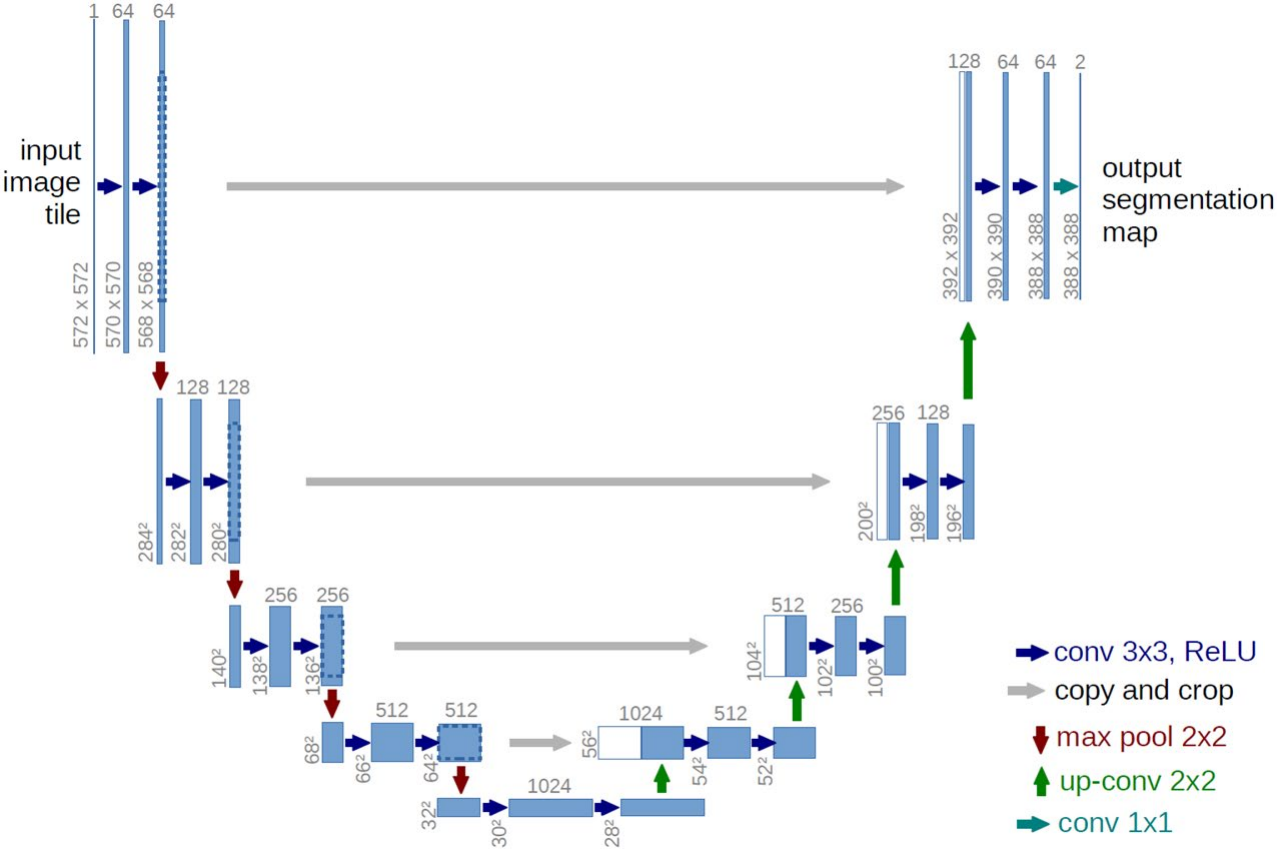
UNet architecture^36^.

For the image segmentation task, the popular architecture is UNet which was originally used in biomedical image segmentation^35^. The architecture contains a collection of convolution blocks for downsampling and upsampling layers where each downsampling and corresponding upsampling layer are connected by the skipped connections (the grey line) in Fig. 22.

**Figure 23:**
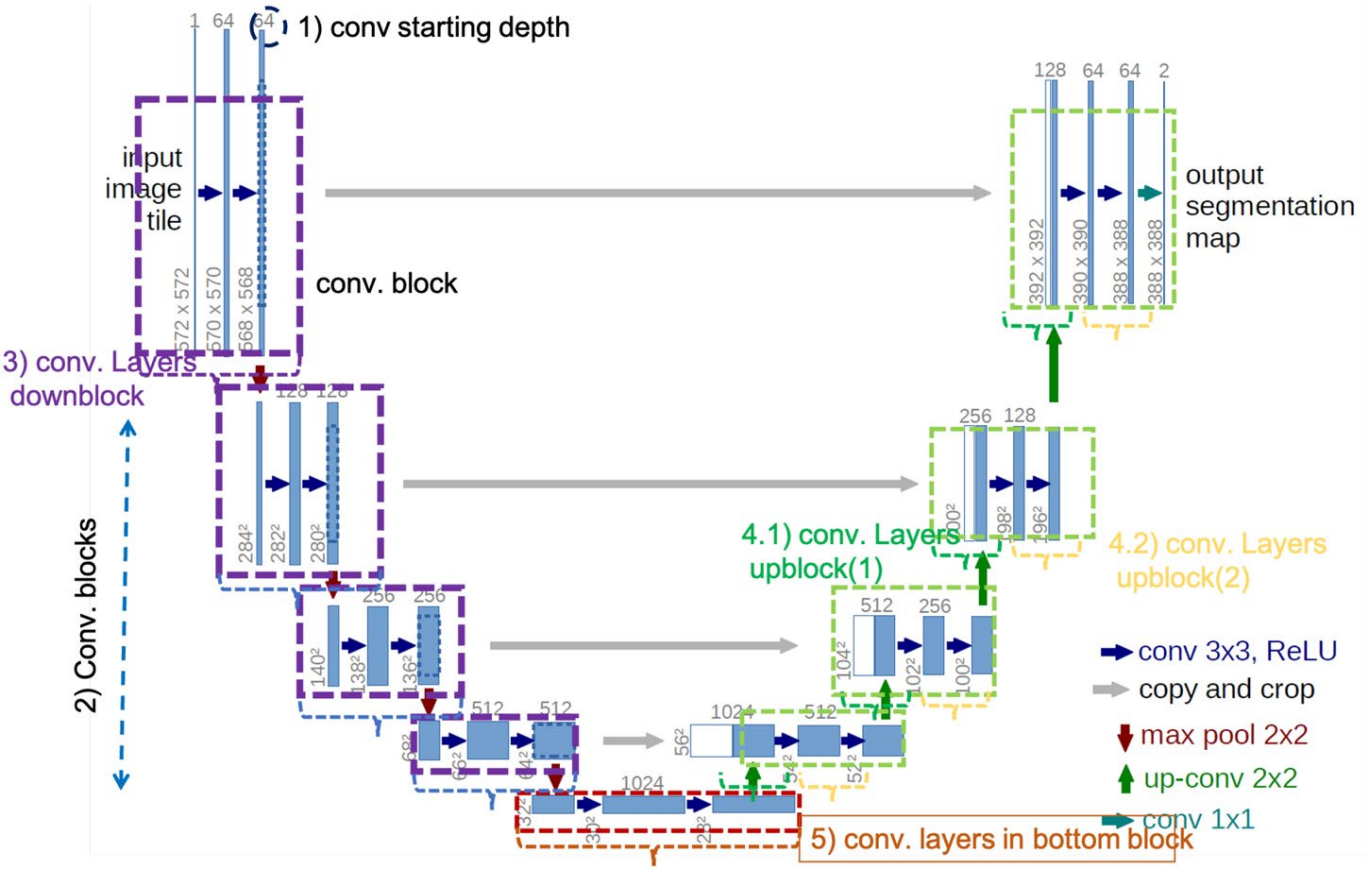
UNet-Auto Template architecture modified from^36^.

From the original architecture, the micro-architecture can be customized as follows (Fig. 23). 1) the starting depth of the convolutional layers 2) the number of convolutional blocks to/from the bottom (the number of the purple/green dashed boxes) 3) the number of layers in each down-block 4) the number of layers in each up-block which is divided into 4.1) the number of layers in each up-block (part 1) 4.2) the number of layers in each up-block (part 2) 5) the number of convolutional layers in the bottom block.

The hyperparameters explored are dropout rate, optimization method, learning rate, etc. Table 11 presents the hyperparameters and micro-architecture parameters along with machine learning parameters that are integrated into our template design for Fig. 23. The default values are chosen in the same manner as the original architecture.

**Table 11:** Hyperparameters and micro-architecture parameters for UNet-Auto.

Parameters	Possible values
Learning rate	0.001, 0.0001, 0.0001 (default=0.001)
Optimizer	RMSprop, SGD
Dropout	[0.1,0.5,0.8] (default=0.5)
# Convolution starting depth	8,16,32 (default=16)
# Convolution layers in each down block	[1–5] (default=3)
# Convolution layers in each upblock (1)	[1–5] (default=1)
# Convolution layers in each upblock (2)	[1–5] (default=2)
# Convolutional blocks	3,4,5,6 (default=4)
# Convolution layers in the bottom block	2,3,4,5,6 (default=2)
Loss function	Categorical_crossentropy, dice_coef_loss

**Figure 24:**
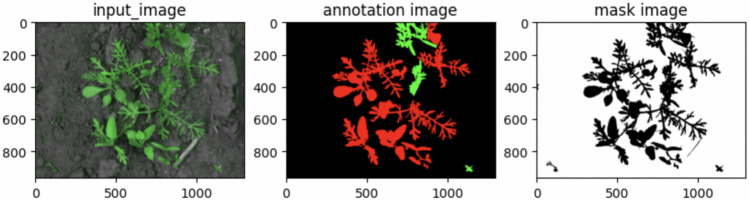
Weed dataset.

The segmentation task was applied to weed dataset^37^ (https://github.com/cwfid/dataset/archive/v1.0.tar.gz). The dataset contains 59 images with annotation and masking as in Fig. 24.

Table 12 presents the top 5 ranks of the discovered models for each algorithm based on the validation accuracy. The training was done for 150 epochs and 100 trials. Compared to the traditional model in Fig. 22 which achieves 0.964 accuracies, UNet-Auto can achieve higher accuracy (0.977, 0.973, 0.974) for all search algorithms. The model sizes vary due to the number of convolutional blocks and the number of convolutional layers inside each block. From the exploration, one can choose the proper model configuration with acceptable accuracy. This shows the effectiveness of finding the proper configuration from the baseline network.

**Table 12:** Model size and accuracy for UNet-Auto.

Rank	UNet-Auto					
	Random		Hyperband		Baysian	
	Model size	Acc	Model size	Acc	Model size	Acc
1	6,082,307	0.977	2,334,467	0.973	3,878,019	0.974
2	2,924,547	0.975	3,500,291	0.973	39,121,347	0.972
3	6,094,595	0.971	3,500,291	0.962	39,121,347	0.972
4	37,351,107	0.967	2,334,467	0.962	6,687,651	0.967
5	6,687,651	0.933	3,514,627	0.959	4,327,331	0.959

Table 13 presents the configurations of top-3 solutions (r1, r2, r3) for each search algorithm. Among all these, the best model achieves more parameters than those of the baseline. However, if one prefers the small model, the model from the Bayesian algorithm (r1) yields the equivalent solution with more accuracy. The minimum convolutional depth (8) can be used to achieve the equivalent or better accuracy while lengthening the number of convolutional blocks. All results show that RMSprop is a proper optimization for this task. Such a template can help designers explore micro-architecture options along with hyperparameter choices effectively.

**Table 13:** Model configuration for UNet-Auto (various search algorithms).

Parameters	Orig	Random			Hyperband			Baysian		
		r1	r2	r3	r1	r2	r3	r1	r2	r3
Learning rate	0.001	0.0001	0.001	0.01	0.01	0.001	0.001	0.01	0.0001	0.0001
#conv_depth	16	32	32	32	32	16	16	8	32	32
#conv_block	4	4	4	5	3	3	3	3	6	6
#conv_layer (down)	3	4	4	4	5	2	2	5	5	5
#conv_layer (up1)	1	1	2	1	1	3	3	1	1	1
#conv_layer (up2)	2	1	5	2	3	3	3	1	2	5
#num_conv_bottom	3	5	3	5	2	5	5	6	6	6
Optimization	RMS	RMS	RMS	RMS	RMS	RMS	RMS	RMS	RMS	RMS
Dropout	0.5	0.8	0.5	0.5	0.8	0.5	0.5	0.8	0.8	0.8
Loss	cat	cat	cat	cat	cat	cat	cat	cat	cat	cat
#parameters	2.3M	6.0M	2.9M	6.0M	2.3M	3.5M	3.5M	2.3M	3.5M	3.5M
#accuracy	0.964	**0.977**	**0.975**	0.971	0.973	0.973	0.962	** 0.974**	0.972	0.972

Significant values are in [bold].

### Object detection

The code from^38^ deploys MobileNetV2^39^ with Single Shot Detector (SSD)^40^ as a backbone is adopted. We implement the variation on the micro-architecture on it. The original MobileNetV2 architecture composes of 7 bottleneck layers (B1_1 to B7_1) where a total number of parameters is 2.2M as shown in Table 14. The backbone SSD^40^ consists of 4 convolutional layers and utilizes MobileNetV2 to extract features.

**Table 14:** MobileNetV2 architecture^38^.

Layers	size
InputLayer	( 224, 224, 3)
Conv2D	( 111, 111, 32)
BatchNormalization	( 111, 111, 32)
ReLU	( 111, 111, 32)
Bottleneck_B1_1	( 111, 111, 16)
Bottleneck_B2_1	( 56, 56, 24)
Bottleneck_B2_2	( 56, 56, 24)
Bottleneck_B3_1	( 28, 28, 32)
Bottleneck_B3_2	( 28, 28, 32)
Bottleneck_B3_3	(28, 28, 32)
Bottleneck_B4_1	(14, 14, 64)
Bottleneck_B4_2	( 14, 14, 64)
Bottleneck_B4_3	( 14, 14, 64)
Bottleneck_B4_4	(14, 14, 64)
Bottleneck_B5_1	( 14, 14, 96)
Bottleneck_B5_2	( 14, 14, 96)
Bottleneck_B5_3	( 14, 14, 96)
Bottleneck_B6_1	( 7, 7, 160)
Bottleneck_B6_2	( 7, 7, 160)
Bottleneck_B6_3	( 7, 7, 160)
Bottleneck_B7_1	(7, 7, 320)
Conv2D	( 7, 7, 1280)
AveragePooling2D	( 1, 1, 1280)
Conv2D	( 1, 1, 11)

**Figure 25:**
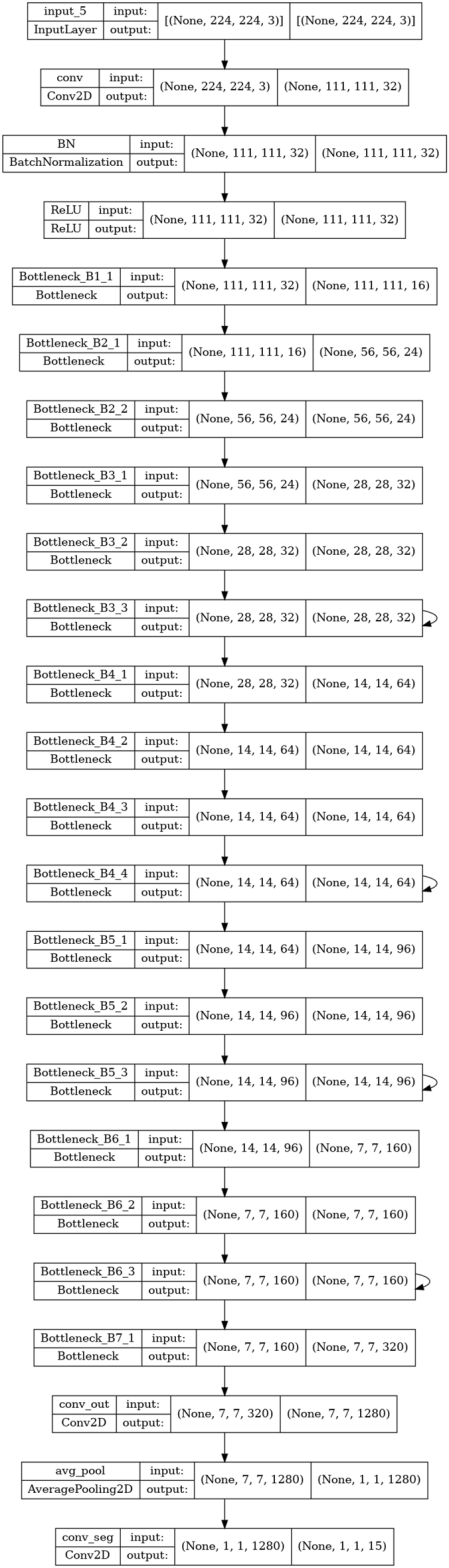
MobileNetV2-Auto.

We duplicate the convolutional layers for bottleneck blocks in MobileNetV2, e.g., B3_3,B4_3,B5_3, and B6_3. Note that the bottleneck block is based on depthwise separable convolution^39^, duplicating the convolutional layers can lead to a deeper network without enlarging the number of parameters^41^. The template architecture is shown in Fig. 25. The cycles in the four layers represent the replications. The number of possible replications is set within [1, 2, 3] where the default value is 1. Other than that, we vary the learning rate parameters in the same way as in the previous experiment.

**Figure 26:**
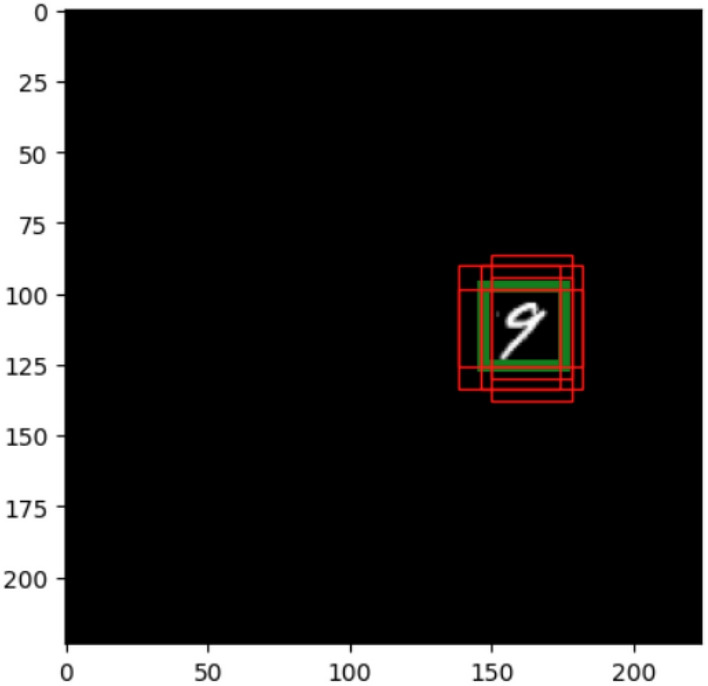
MNIST dataset with bounding box.

The MNIST dataset with object detection bounding box is generated as in the example in Fig. 26. Confidence loss and SmoothL1 loss are used as a metric. As in the original code, the training data size was 600 and the testing data size is 100.

Table 15 shows the validation loss for each found model for the different search algorithms. We show only the validation loss since all model sizes are the same as the original one. The original model yields a loss value 0.01 while all our cases can lead to a smaller loss.

**Table 15:** Validation loss for various MobileNetV2-SSD-Auto.

Rank	MobileNetV2-SSD- Auto Loss		
	Random	Hyperband	Bayesian
1	0.0073	0.0072	0.0070
2	0.0075	0.0074	0.0075
3	0.0078	0.0081	0.0076
4	0.0080	0.0083	0.0083
5	0.0083	0.0083	0.0084

Table 16 lists the replication (*rep*) configuration found for the cases of the top three loss values. Varying the number of convolutional layers can lead to more accuracy while not increasing the model size. Thus, utilizing such a template model enables the exploration of micro-architecture choices which can improve the model’s effectiveness.

**Table 16:** Model configuration for MobileNetV2-SSD-Auto (various search algorithms).

Parameters	Orig	Random			Hyperband			Baysian		
		r1	r2	r3	r1	r2	r3	r1	r2	r3
learning rate	0.01	0.0001	0.0001	0.01	0.01	0.0001	0.01	0.0001	0.0001	0.0001
B3_1 rep	1	2	3	2	3	2	3	3	3	3
B4_1 rep	1	1	2	3	2	3	1	3	1	3
B5_1 rep	1	2	1	1	2	3	3	1	3	1
B6_1 rep	1	2	1	2	1	1	2	2	3	2
Loss	0.01	**0.0073**	0.0075	0.0078	**0.0072**	0.0074	0.0081	**0.0070**	0.0075	0.0076

Significant values are in [bold].

## Conclusion and future work

In this paper, we propose a framework for exploring model choices based on AutoModel. The baseline model can possibly be attached with modules such as SE block, skip connection, etc. The proper number of components is selected while the model hyperparameters can be searched at the same time. Better model architectures are possible to be found with smaller network sizes. First, we demonstrate the approach based on the SqueezeNet model attaching the SE block with skip connections at variable positions. Such a model is recognized as a template model. The variable lengths and attachment points can be explored with the standard search algorithms: Random, Hyperband, and Bayesian. The models with higher accuracy are obtained when attaching a small number of SE blocks and skip connections automatically.

We also present the alternatives of applying micro-architecture variation for the other models for different tasks such as image segmentation and object detection. For the segmentation task, the UNet-Auto template can vary many parts such as the number of convolutional blocks and number of convolution layers and depth, etc. For object detection, the MobileNetV2-SSD-Auto template can vary the number of convolutional layers for each bottleneck. With such a template, the model structure with the best accuracy and size can be obtained conveniently.

The future work will include the mechanism of considering customized search schemes.

## Data Availability

The source code is available at the author Github https://github.com/cchantra/SqueezeNetSEMAuto.
